# Cooking, cleaning, and tossing: high-resolution analysis of domestic activities at the Mid-Neolithic site of Molino Casarotto (Vicenza, NE Italy)

**DOI:** 10.1007/s12520-025-02353-w

**Published:** 2025-12-11

**Authors:** Cristiano Nicosia, Gregorio Dal Sasso, Federico Polisca

**Affiliations:** 1 Department of Geosciences, University of Padova (IT), Padova, Italy; 2 Institute of Geosciences and Earth Resources, National Research Council of Italy, Padova, IT Italy

**Keywords:** Neolithic, Molino Casarotto, Shell middens, Hearths, Lacustrine setting, Micromorphology, Geoarchaeology

## Abstract

The Middle Neolithic lakeshore site of Molino Casarotto (4700 − 4400 cal BCE; Vicenza province, northern Italy) preserves some of the most informative deposits related to Neolithic daily life in Italy. The organisation and use of space within the Neolithic domestic contexts remain poorly understood, largely due to the limited preservation of archaeological evidence. At Molino Casarotto, however, these aspects can be explored thanks to the presence of domestic hearth rake-out layers and shell middens, which form substantial accumulations interdigitating with repeatedly renovated cooking plates. Several heated stones were recovered in association with these combustion structures. High-resolution sediment analyses (micromorphology, micro-FTIR, and XRD) allowed for the characterisation of the sediments used to construct the cooking plates, the reconstruction of combustion temperatures, and gave insights into mollusc cooking techniques. In particular, the discarded shells were likely boiled or roasted at low temperatures (< 200 °C), as indicated by the absence of microstructural alterations and the preservation of aragonite, possibly using heated stones. In contrast, thermally altered shells embedded in ash layers suggest exposure to post-depositional heating events. Eventual isotopic analyses should therefore take into account the taphonomic history of the specimens, as differential preservation may occur even across short distances. Finally, although structural remains at the site were limited, microarchaeological evidence indicates that the cooking plates were used within a sheltered area characterised by rapid and continuous sediment accumulation, with no evidence of abandonment.

**Supplementary Information:**

The online version contains supplementary material available at 10.1007/s12520-025-02353-w.

## Introduction

In intra-site archaeological contexts, geoarchaeological investigations proved their significance in the reconstruction of past human behaviours and living conditions, allowing us to characterise both social and cultural aspects of ancient communities. Sedimentary deposits have been profitably used for this purpose, as anthropogenic sediments can be intended as ‘micro-artefacts’ derived from human activities (Polisca 2025 and references therein). Compared to macroscopic artefacts, which tend to be removed (Watson 1979, p. 157; Wilk and Schiffer 1979, p. 197; Miller Rosen 1986, p. 92; Tani 1995, p. 246), sediments more likely survive daily activities in their primary depositional setting (‘residual refuse’ in Schiffer 1987, p. 62; see also La Motta and Schiffer 1999, p. 21; Milek 2012, p. 125). Even when they are dumped (‘secondary refuse’ in Schiffer 1987), they can still provide significant information on the activities carried on nearby (Simpson and Barrett 1996; Schiegl et al. 2003; Shillito et al. 2011; Nicosia et al. 2022; Marcazzan et al. 2022; Tomé et al. 2024).

Unfortunately, Neolithic sites in the Po Plain (northern Italy) are typically characterised solely by negative features (*sensu* Harris 1989, p. 47) and their infilling, with no other evidence of accumulation. In the region, the absence of anthropogenic deposits is largely due to the limited aggradation that characterised the Late Glacial Maximum alluvial megafans – where most Neolithic sites are located – that derives from the entrenchment of the main rivers within the Late Pleistocene/Holocene floodplain (Cremaschi 1990; Biagi et al. 1993; Fontana et al. 2014). As a result, Neolithic occupation layers were often disturbed by later human activities such as ploughing, leading to the irreparable loss of the archaeological deposits (Degasperi 2000; Cavulli 2008a, pp. 103–106). In most sites the analysis of pit infillings has played a crucial role in interpreting their function (e.g., refuse pits, hearths within shallow pits, storage pits, pit-dwellings, postholes; see Degasperi 2000, pp. 6–8 for a synthesis), as well as in reconstructing activity areas (Pessina 1998; Degasperi 2000; Bernabò Brea et al. 2008; Cavulli 2008a, b; Pearce 2008; Pessina and Tiné 2022).

**Fig. 1:**
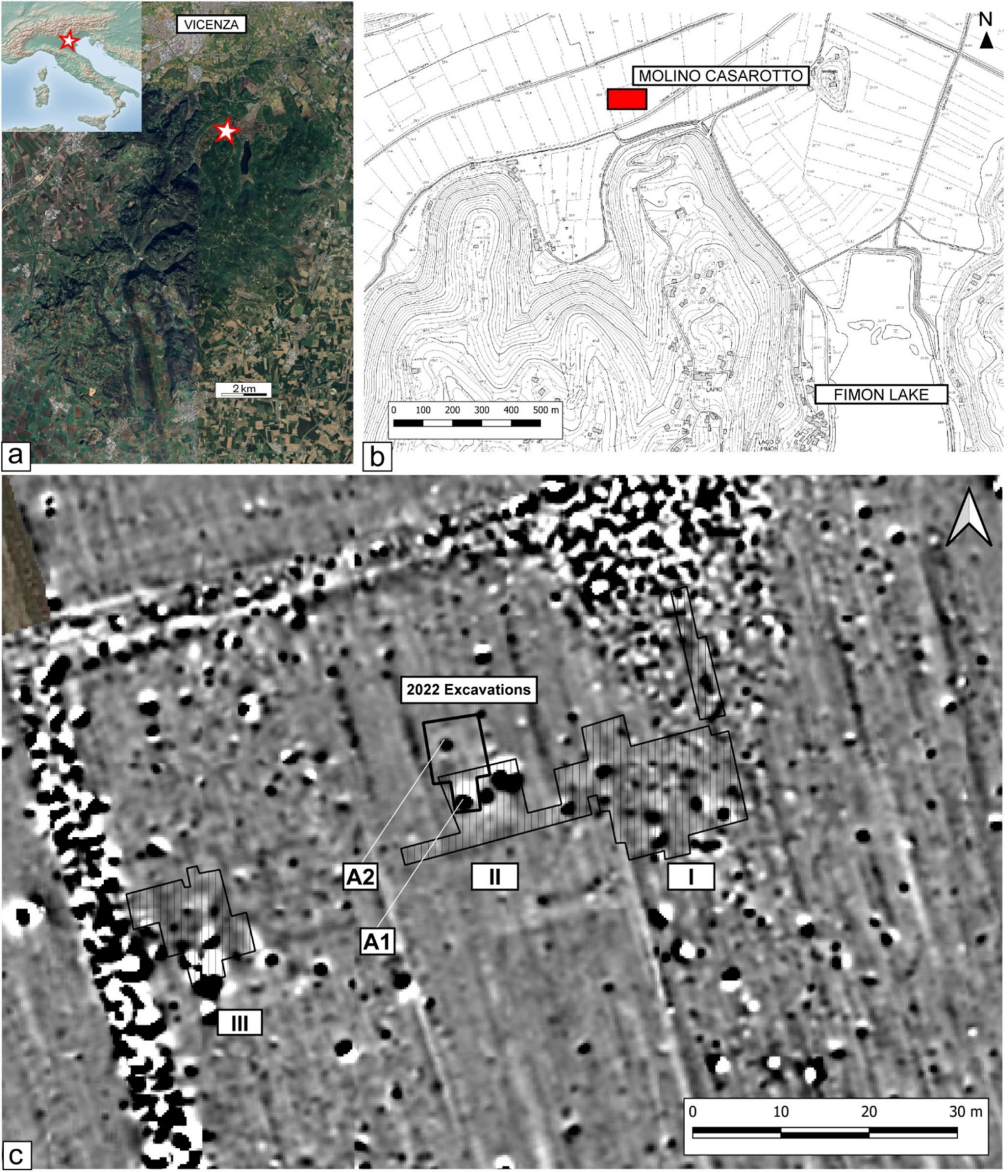
Site location, investigated areas, and results of magnetometric surveys: **a-b**) position of the site of Molino Casarotto in the ‘Valli di Fimon’ area (Berici Hills). Image in the top left corner was obtained from www.naturalearthdata.com and is public domain (modified from Nicosia et al. 2025, fig. 1); **c**) results of magnetometric surveys with: I, II, III – first, second, and third inhabitation areas of the 1969–1972 excavations (see Barfield and Broglio 1986); A1 – magnetic anomaly related to the complex fireplace of the 1969–1972 excavations; A2 – magnetic anomaly related to the new fireplace exposed during the 2022 excavations (modified from Nicosia et al. 2025, fig. 2)

Within this context, a notable exception is the Middle Neolithic site of Molino Casarotto (ca. 4700 − 4400 cal BCE, see Nicosia et al. 2025), located south of Vicenza in the Veneto region at the foot of the Berici Hills (Fig. 1a). Here, some of the best-preserved domestic deposits of the Italian Neolithic have been discovered, featuring complex stratigraphic sequences with combustion structures, rake-out layers, and shell middens (see section The site of Molino Casarotto).

**Fig. 2:**
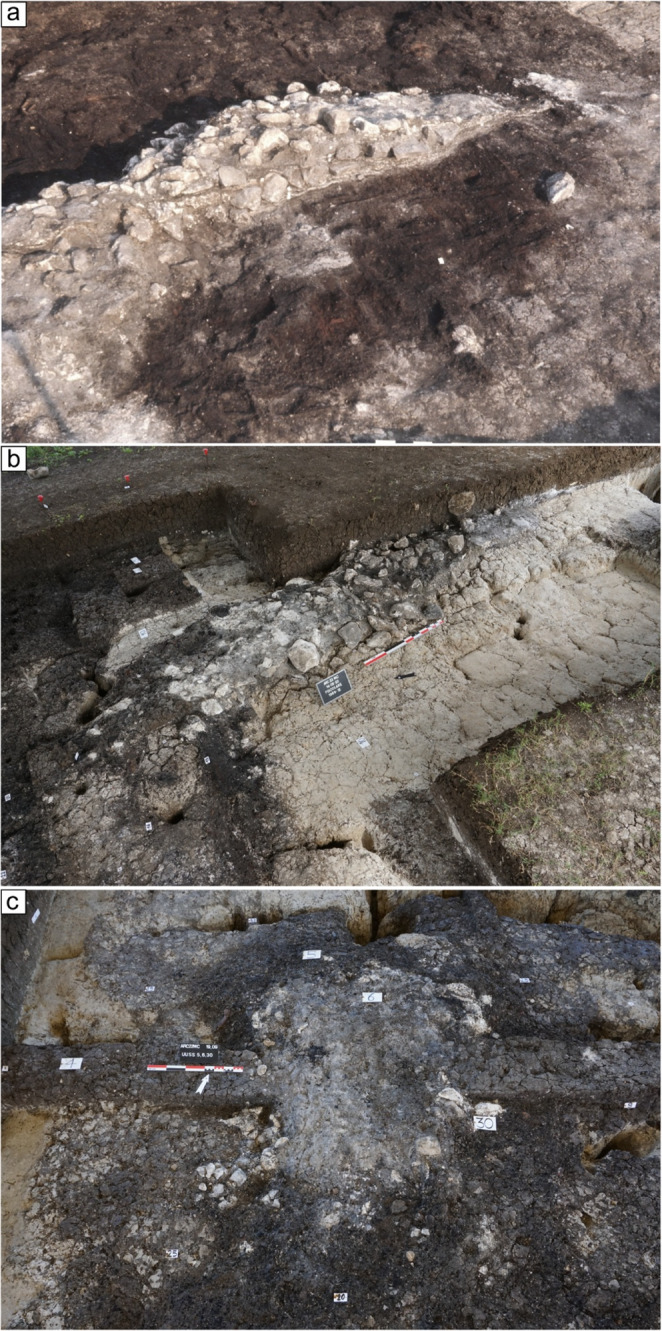
Field images: (**a**) 1972 field image showing the complex hearth with several stones. Note the presence of a wooden platform underneath. For a clearer picture of the profile view, see Fig. 4; (**b**) same as ‘a’, but in the 2022 excavations. Note the complete decay and disappearance of the wooden platform visible in ‘a’; (**c**) ash deposit accumulated on top of the new rounded cooking plate exposed in the 2022 excavations (see Fig. 7). Note the presence of stones delimiting the feature

Through an extensive micromorphological analysis and additional chemical and mineralogical analysis of both in situ hearths and secondary deposits (i.e., hearth rake-out layers and shell middens), this geoarchaeological investigation will therefore:

1. Reconstruct the daily practices at the site related to the use of the combustion structures.
2. characterise the materials employed for their construction;
3. complement the available archaeobotanical and archaeozoological data on subsistence strategies, and cooking techniques at the site, with particular attention to molluscs (Breglia et al. 2025 and references therein).

In addition, since architectural evidence at the site is scarce (see section The site of Molino Casarotto), this paper also aims to use micromorphology to determine if the sedimentation occurred indoors or outdoors. Finally, this high-resolution approach also allowed us to assess the continuity of occupation at the site and to identify possible phases of abandonment.

### The site of Molino Casarotto

The site of Molino Casarotto, pertaining to the Squared-Mouth Pottery culture, was a peri-lacustrine settlement close to the Fimon Lake (Barfield and Broglio 1986). Archaeological excavations between 1969 and 1972 and in 2022 unearthed complex stratigraphic sequences of three inhabitation areas (“*aree di abitazione*” in Bagolini et al. 1973), preparation layers (‘*bonifica*’ in Italian) made with wood branches (Fig. 2a), and multi-stratified hearths intercalated with shell middens and hearth rake-out layers (Fig. 2a-b; Barfield and Broglio 1986; Nicosia et al. 2025). At Molino Casarotto, hearths can be defined as constructed cooking plates (‘*piastre di cottura*’ in Italian; see section Neolithic combustion features in Italy). These consist of selected clay or silty clay sediments laid on the ground to form rounded to rectangular structures with flat or slightly convex surfaces (Fig. 2c; cf. Cavulli 2008b, p. 87; Degasperi et al. 2019, p. 3; Tasca et al. 2019, p. 21). These structures were built directly on the ground or, more frequently, on wooden platforms (Bagolini et al. 1973). They often underwent multiple renovation episodes, with new sediment layers laid directly atop earlier cooking plates or their related rake-out deposits. In many cases, cooking plates were covered or surrounded by numerous heated stones, defining the perimeter of the structures (Bagolini et al. 1973; Nicosia et al. 2025). Shell middens were often associated with cooking plates and deliberately piled to their south (Barfield et al. 1986, p. 69; Nicosia et al. 2025). Considering the exceptionality of such evidence, a geoarchaeological investigation of these anthropic features can greatly enhance our understanding of daily life and choices of Neolithic communities in northern Italy.

**Fig. 3:**
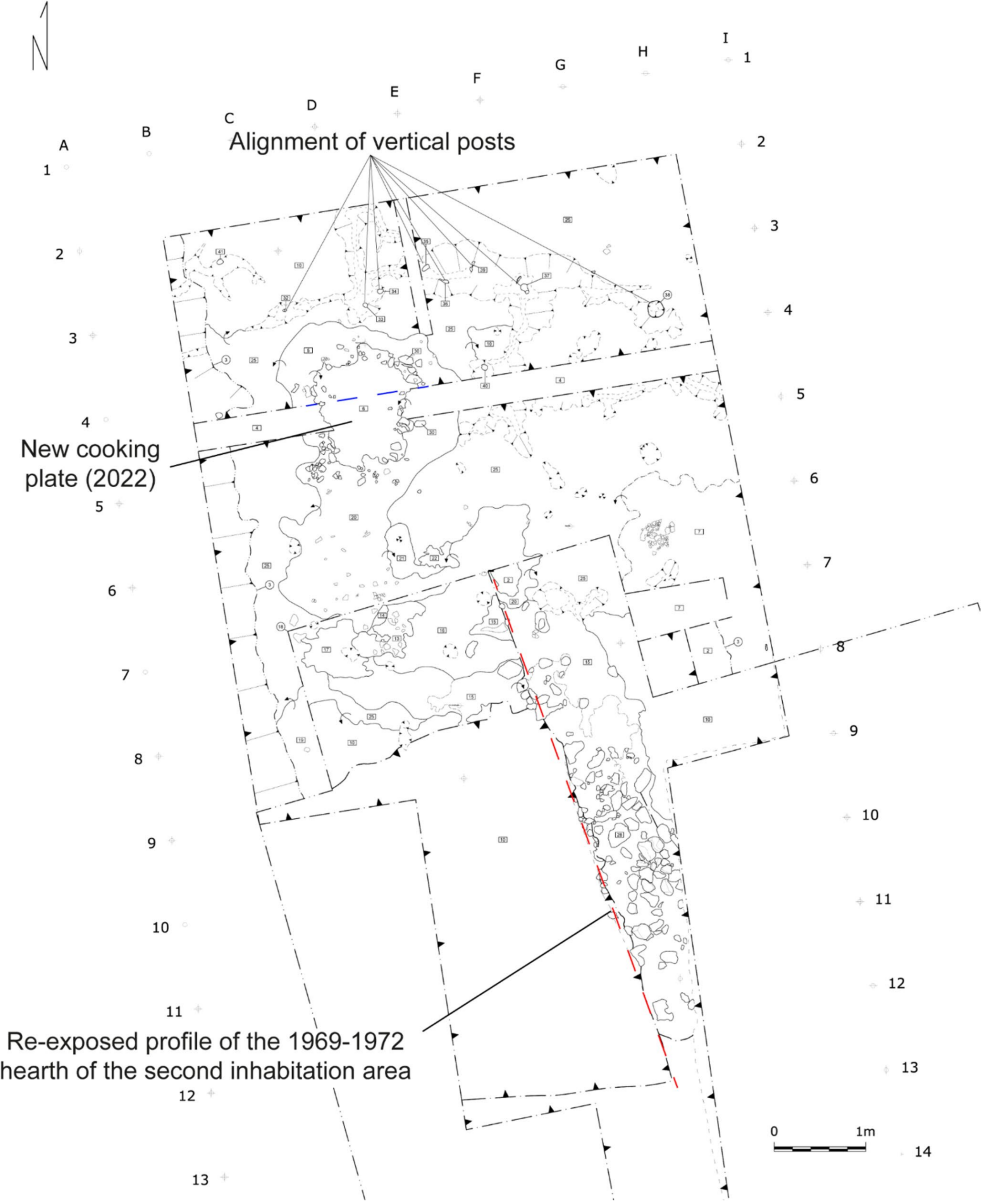
Excavation plan with the 2022 investigations. The main features discussed in the article are indicated (modified from Nicosia et al. 2025, fig. 3).The red dashed line indicates the location of the profile shown in Fig. 4. The dashed blue line, instead, indicates the profile shown in Fig. 7

Despite the recovery of numerous vertical posts, no clear structural limits were identified in any of the inhabitation areas. During the 2022 excavation, an alignment of vertical posts was discovered delimiting, to the north, an area where a cooking plate was located (Fig. 3), but the field evidence was not sufficient to determine whether this space was sheltered/roofed (Nicosia et al. 2025).

**Fig. 4:**
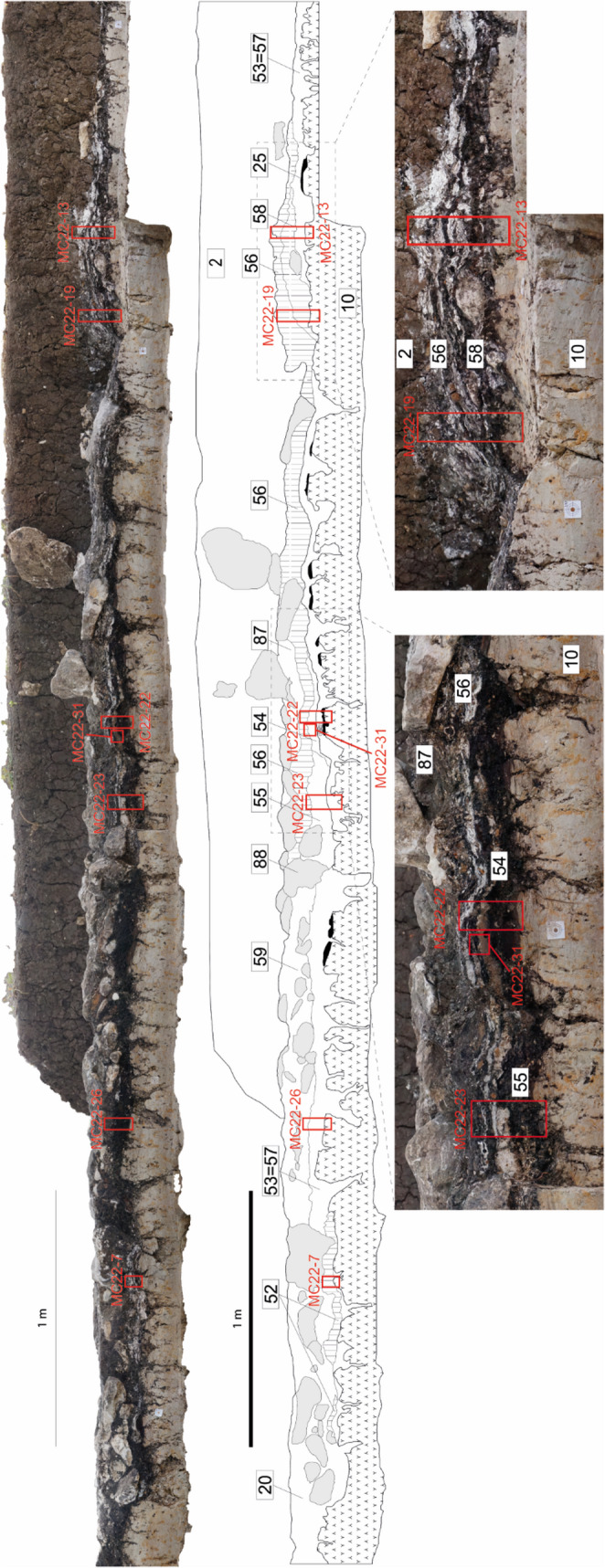
Stratigraphic profile from the 1968–1972 excavation of the complex hearth of the ‘second inhabitation area’. It features a sequence of cooking plates, related rake-out deposits and shell middens which were originally exposed during the 1969–1972 excavations and exposed again in 2022 in the southern portion of the excavation (modified from Nicosia et al. 2025, fig. 6). In red, the position of the micromorphology samples

### Neolithic combustion features in Italy

Neolithic combustion structures typically consist of shallow pits (known as ‘*fossa di combustione*’ in Italian) with rubified walls and an infill comprising charcoal, stones, and wooden elements (Cavulli 2008a, pp. 321–322; Costa et al. 2019, p. 28). A distinctive group of these features includes a layer of charcoal and larger wooden elements covered by heat-fractured stones (Cavulli 2008a, p. 322; Pessina and Tiné 2022). Depending on the context, these structures, variously referred to as ‘earth ovens’ (Thoms 2008), ‘*empierrements*’ or ‘*roches chauffées*’, ‘*foyers/fosses à blocs de pierres*’ (Gascó 2002, pp. 15–16), *‘four*/*foyer polynésien*’ (French), or ‘*forno polinesiano’* (Italian), have been interpreted as being linked to pottery production (Sarti et al. 1991, p. 86; Costa et al. 2017), ritual activities (Marzatico et al. 2019), or meat cooking (Beeching 2017; Pescio et al. 2017). The latter interpretation is supported by ethnographic and geo-ethnoarchaeological comparisons (Métraux 1935; Orliac and Orliac 1980, 1982; Orliac and Wattez 1989; Thoms 2003, 2008), which suggest that these structures functioned as indirect cooking systems, allowing for prolonged and controlled heating through the use of refractory materials (Foucher et al. 2000, p. 190; Costa et al. 2019, p. 28). As previously mentioned, at Molino Casarotto, clusters of heat-altered pebbles and blocks were found in association with hearths. However, this evidence differs from the combustion pits with *empierrements*, as no negative features were identified.

In the Italian Neolithic, simple hearths are also documented, with fires typically made directly on the ground without any surface preparation. These are identified by their rubified surfaces with circular to sub-elliptical shapes and lack any stone delimitation (Cavulli 2008b, p. 87, a, p. 321). They differ from the aforementioned constructed cooking plates, which involve the intentional laying down of selected sediment to create an earthen structure (Cavulli 2008b, p. 87; Degasperi et al. 2019, p. 3; Tasca et al. 2019, p. 21). In such cases, cooking activities could also take place through heat conduction by the surface of the plate (Gascó 2002; Peinetti et al. 2023). As anticipated, similar cooking plates were identified at Molino Casarotto, typically circular in shape, although rectangular examples have been documented (Barfield et al. 1986, p. 69; Nicosia et al. 2025, pp. 57–58). Since numerous cooking plates at Molino Casarotto were built atop wooden platforms, it is also possible that, in some cases, they functioned as simple hearths with the preparatory layer serving only to insulate the platform from the fire (cf. Gascó 2002; Cattani et al. 2015; n. 9). While ovens and furnaces are known in the Italian Neolithic (e.g., Cavulli 2008a, p. 324; Conati Barbaro et al. 2019; Costa et al. 2019, p. 42; Degasperi et al. 2019), no similar features were observed at Molino Casarotto.

### Neolithic shell middens in Italy and the relevance of a geoarchaeological approach

Despite the significance of molluscs in Italian Neolithic subsistence (Barker et al. 1987, p. 107), shell middens have rarely been identified. Mollusc shells are usually found within pits or scattered in archaeological layers, often in association with pottery remains, lithics, charcoal, charred fruits, seeds, and animal bones (Girod and Starnini 2022; Minniti 2005 and references therein).

Only in a few cases concentrations of mollusc shells were documented, mainly in marine coastal areas (Deith 1987; Wilkens 1997; Melis et al. 2021) and/or in Mesolithic-Early Neolithic contexts (Mannino et al. 2007; Colonese et al. 2014). Rare lacustrine settlements have yielded significant evidence of shell middens, such as La Marmotta (Bracciano Lake, Lazio region; Fugazzola Delpino et al. 1993, p. 190) and, as mentioned, Molino Casarotto. At Molino Casarotto, shell middens consist almost exclusively of *Unio* sp. shells (Breglia et al. 2025), likely attributable to *Unio* cf. *elongatulus* C. Pfeiffer, 1825 (Girod and Starnini 2022, p. 6; Marrone et al. 2019).

As noted by Robertshaw et al. (1983, p. 6), stratigraphic units “based on shells are often likely to be of very limited areal extent. Shells from two sequential days of shellfish gathering and processing dumped in different areas of the midden may form very different archaeological horizons as a result of factors such as the numbers and species of shellfish collected, variations in processing activities, whether ash or other rubbish was discarded with the shells, and the degree of subsequent post-depositional trampling”. Geoarchaeological methods have thus often been employed to unravel the complexity of gestures (*sensu* De Beaune 2000) involved in the formation of shell middens, with micromorphology playing a key role in providing contextual information (e.g., Villagran et al. 2009, 2011a; Villagran 2014; Aldeias and Bicho 2016; Duarte et al. 2019).

Shells have also been studied to understand the effects of heating on their colour and structure in order to reconstruct combustion temperatures (Spennemann 2004; Maritan et al. 2007; Villagran et al. 2011b; Canti 2017, pp. 183–184; Oertle and Szabó 2023) and, consequently, cooking techniques (Milano et al. 2016; Müller et al. 2017; Aldeias et al. 2019; Staudigel et al. 2019). These experiments demonstrated that boiling does not induce any recognisable signature in shells, except for a mild relaxation in the aragonite lattice (Milano et al. 2016, p. 20; Staudigel et al. 2019, p. 4). Roasting and cooking above 200–300 °C, on the other hand, lead to colour changes which depend on the combustion atmosphere, duration, and temperature (Milano et al. 2016, p. 19, 2018, pp. 446–448; Canti 2017). These variables likely explain the differences in colour changes reported in the literature. Some authors observe a brownish hue emerging around 300–400 °C – probably due to protein combustion (Canti 2017) – which then progressively shifts to greyish and blackish as temperatures rise (Villagran et al. 2011b; Milano et al. 2016; Simões and Aldeias 2022, p. 8; Oertle and Szabó 2023). Others, instead, report creamy tones appearing around 700 °C (Milano et al. 2016, 2018, p. 448; see also Aldeias et al. 2019, tbl. 1).

From a mineralogical perspective, aragonite shells undergo a clear polymorphic transition to calcite when heated to around 400–450 °C (Maritan et al. 2007, p. 531; Milano et al. 2018, p. 448; Simões and Aldeias 2022, p. 8). However, this process likely begins at approximately 250–300 °C, depending on combustion duration and shell species (Milano et al. 2016, p. 19; Müller et al. 2017, p. 3; Milano and Nehrke 2018; Aldeias et al. 2019). Microstructural changes can help narrow combustion temperature ranges. Generally, at 300 °C, heating begins to fissure shells along the laminae boundaries, with an almost complete separation of the inner and outer layers occurring at 400 °C (Villagran et al. 2011b, tbl. 3; Simões and Aldeias 2022, p. 8). Modifications become more evident around 650 °C (Milano et al. 2016), when micrometre-sized intra- and inter-layer pores form. The shape, dimension, and location of these pores depend on the maximum temperature reached before the complete decomposition of calcite (800–850 °C; Maritan et al. 2007). Above 700–800 °C, shells become so fragile that their preservation in archaeological deposits or during sieving processes is unlikely (Spennemann 2004, p. 15).

Recently, combined geoarchaeological methods have enabled the identification of combustion events on top of shell middens which would otherwise have been overlooked (Simões and Aldeias 2022; see also March et al. 2014 for a macroscopic study). This was achieved by recognising areas with shells exhibiting a clear gradient of transition from aragonite/mixed signal of calcite-aragonite to fully calcite shells, thereby demonstrating that this transition occurred in situ due to heating.

A recent experiment tested the effects of trampling on boiled and roasted shells (Kleijne et al. 2024), demonstrating that it induces compaction, snapping, a platy microstructure, and a horizontal preferential orientation of coarse components. The only significant difference between boiled and roasted shells is that the latter fragment into smaller pieces, likely due to their more brittle and cracked structure (Kleijne et al. 2024, p. 6; Oertle and Szabó 2023; Spennemann 2004).

Geoarchaeological and geo-ethnoarchaeological studies on shell middens have also provided insights into discard practices (i.e., dumping areas vs. dwelling areas), as well as the seasonality of human occupation (Villagran et al. 2009, 2011a; Balbo et al. 2010; Aldeias and Bicho 2016; Duarte et al. 2019). In this regard, oxygen isotope analysis from shells can be crucial, especially in marine coastal areas, as it allows to reconstruct sea temperatures at the time of shell formation (Prendergast et al. 2013; Gutiérrez-Zugasti et al. 2015) and the seasonality of shellfish foraging (Burchell et al. 2013; Prendergast et al. 2016; Prendergast and Schöne 2017). However, experimental research demonstrated that heating processes significantly affect δ^18^O_shell_ values, with alterations increasing with temperature (Milano et al. 2016, 2018; Müller et al. 2017). It is therefore essential to identify both visible and macroscopically invisible combustion processes to accurately interpret such analysis.

## Materials and methods

**Fig. 5:**
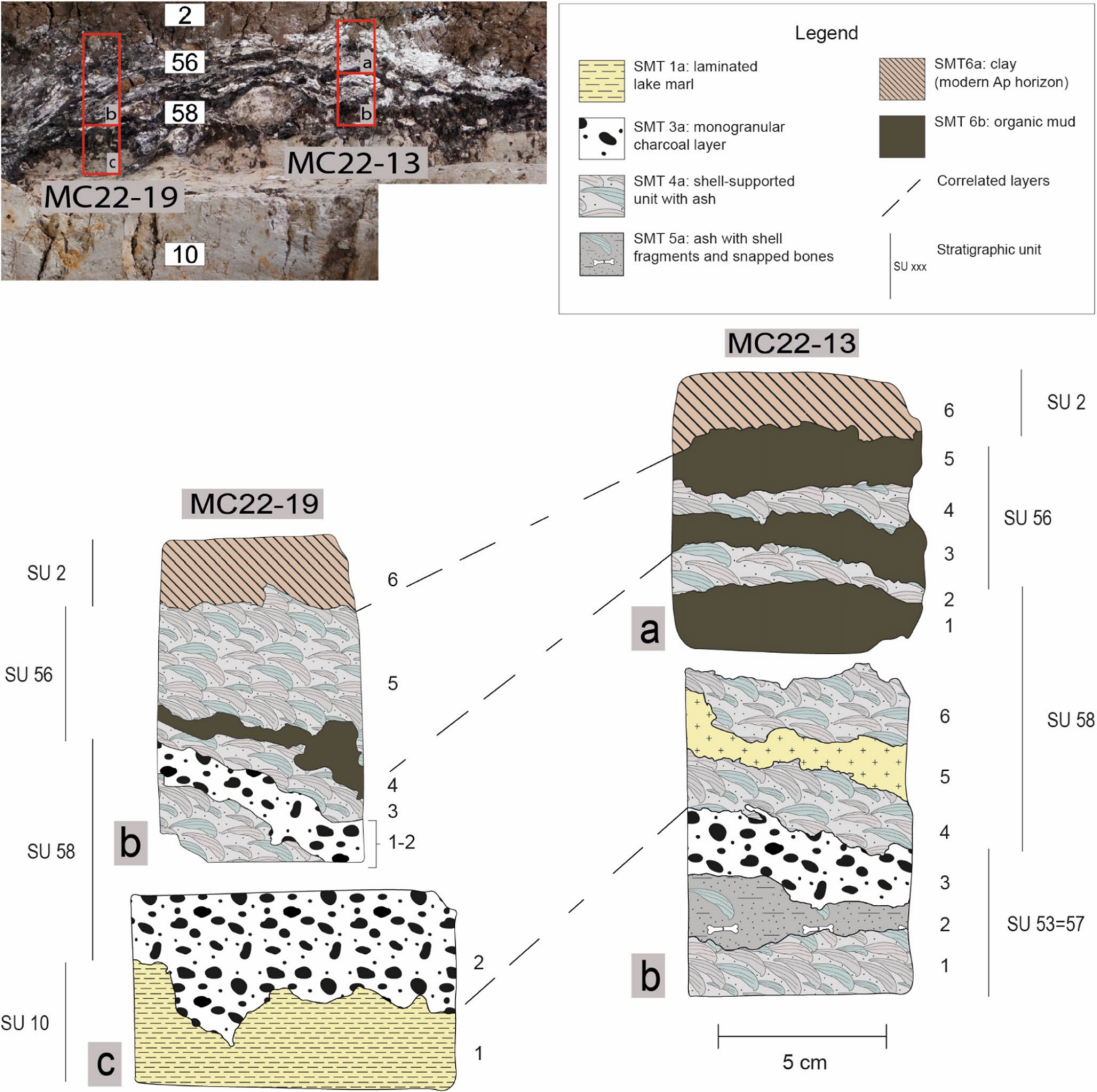
Sampling location and interpretation of the southern portion of the 1969–1972 complex hearth profile. Top: detail from Fig. 4, indicating the position of samples MC22-13 and MC22-19. Bottom: interpretation of the thin sections, with an assigned SMT for each subunit

**Fig. 6:**
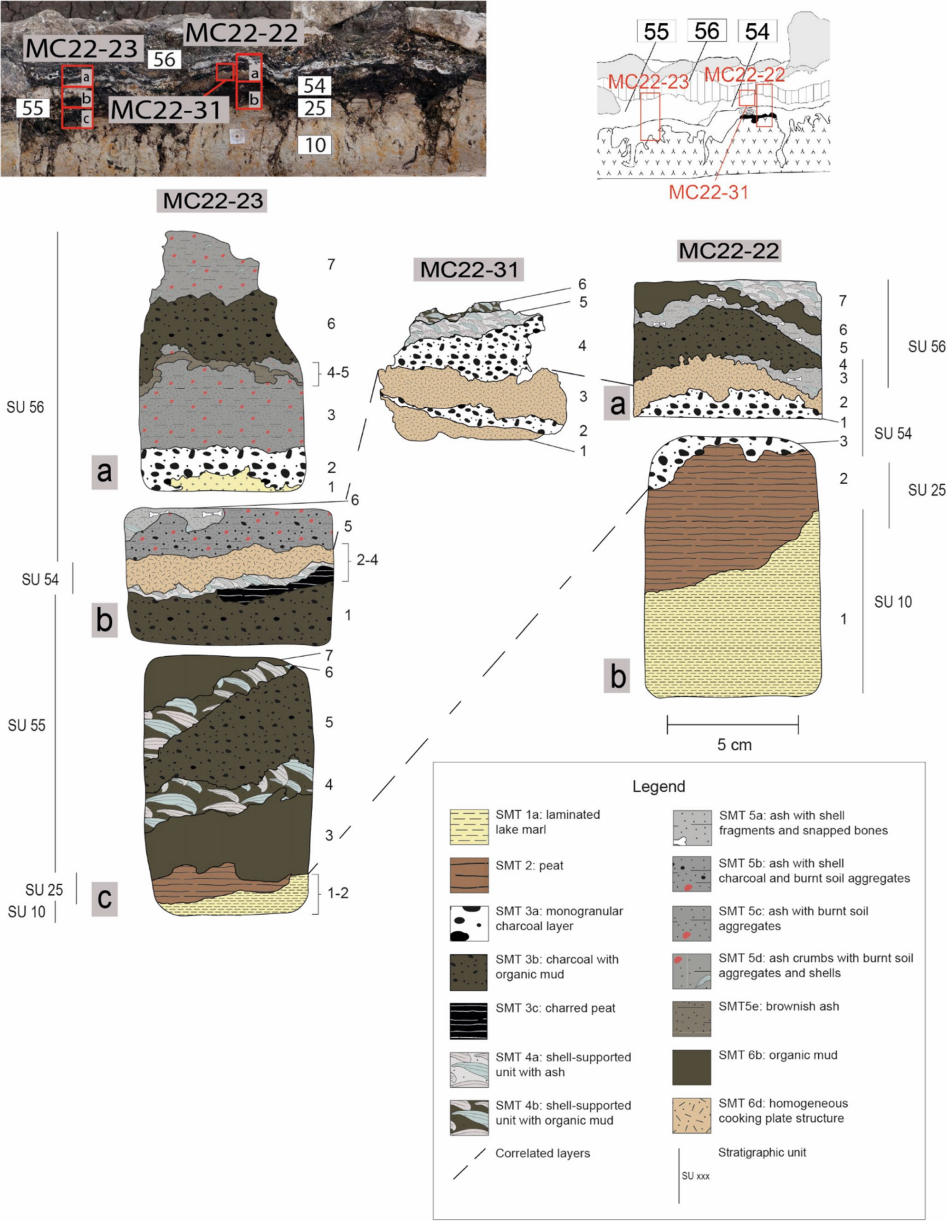
Sampling location and interpretation of the northern portion of the 1969–1972 complex hearth profile. Top: detail from Fig. 4, indicating the position of samples MC22-22, MC22-23, and MC22-31. Bottom: interpretation of the thin sections, with an assigned SMT for each subunit

**Fig. 7:**
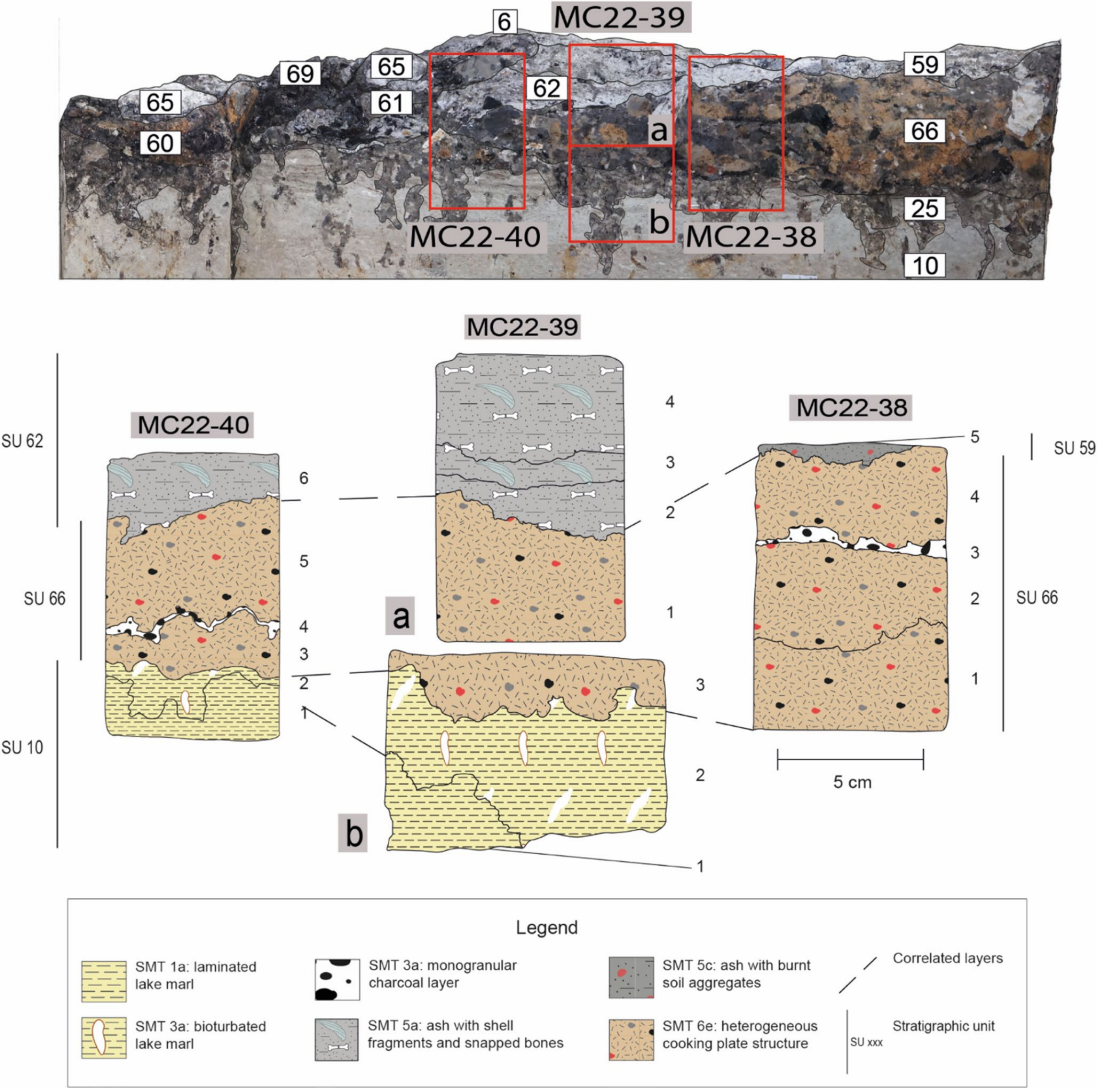
Sampling location and interpretation of the vertical accretion related to the cooking plate discovered in 2022. Top: detail of the profile indicated in Fig. 3, showing the position of samples MC22-38, MC22-39, MC22-40. Bottom: interpretation of the thin sections, with a SMT assigned to each subunit

The 2022 excavation was conducted in open area, following the stratigraphic method (Harris 1989), describing morphology, texture, and composition of each stratigraphic unit (hereafter, SU). Temporary baulks were left unexcavated for micromorphological sampling (Stoops and Nicosia 2017). In particular, sampling involved:

1. the entire sequence of the 1969–1972 complex hearths with cook-stones and shell middens (Figs. 5 and 6);
2. the new cooking plate uncovered in 2022 (Fig. 7).

Micromorphological sampling was coupled with additional bulk samples collected from each stratigraphic unit and macroscopically visible soil aggregates composing the cooking plates (see section The new cooking plate from the 2022 excavation).

### Soil and sediment micromorphology

Micromorphological samples were air dried (i.e., no acetone replacement) and prepared following the methods of Murphy (1986). Thin section analysis was done using in plane polarised light (PPL), cross-polarised light (XPL), and observing the autofluorescence when excited with blue light (BLF). The micromorphological description followed the terminology of Stoops (2021). The identification of components was based on standard literature (Stoops and Nicosia 2017) and references therein). In total, 24 uncovered thin sections were analysed:

- Sixteen thin sections were made from the 1969–1972 complex hearth profile (the most significant ones are shown in Figs. 4, 5 and 6), in order to cover the entire vertical variability, part of the lateral variability, and all the stratigraphic units documented in the profile.
- Eight thin sections to fully represent the lateral variability and vertical accretion of the cooking plate discovered in 2022 (the most significant ones are shown in Fig. 7).

The complexity of the stratigraphic sequences led to the use of Soil/sediment Microfabric Types (hereafter, SMTs; Macphail and Cruise 2001; Nicosia et al. 2022; Polisca 2025). SMTs group together different units and subunits (i.e., layers invisible to the naked eye) that share similar micromorphological features and, consequently, likely formed through comparable depositional and/or post-depositional processes. In this study, types are distinguished based on their dominant component, while subtypes are defined by the association with minor components or specific fabric characteristics (e.g., compactness, orientation of coarse inclusions). Types are identified by a number (e.g., SMT 1), whereas subtypes are denoted by a lowercase letter (e.g., SMT 1a).

### Powder x-ray diffraction (p-XRD)

Powder-XRD analysis aimed to characterise the construction materials for the hearth structures. Samples were carefully handpicked during fieldwork and include both the cooking plates identified in the 1969–1972 profile as well as the newly identified cooking plate. All aggregates and the matrix were analysed to assess compositional similarities, combustion temperatures, and potential sourcing areas. Additionally, a control sample was collected from the colluvial reddish clay derived from the rubified soil of the Berici Hills to verify if this material was used to build the cooking plates.

The analysis was conducted with a Philips X’Pert Pro diffractometer, equipped with a Co source (40 kV, 40 mA) and Bragg-Brentano parafocusing geometry. The instrument featured a Real-Time Multiple Strip (RTMS) X’Celerator detector. Samples were prepared on zero background Si sample holders, with sample rotation facilitated by a Bragg-Brentano HD© spinner. Data were qualitatively analysed using HighScore (Plus) software (version 4.9, 2020; Degen et al. 2014) and COD-derived search-match database (Gražulis et al. 2009).

### Micro-fourier-transform infrared spectroscopy (micro-FTIR)

Micro-FTIR analyses in attenuated total reflectance (ATR) mode were carried out as point analyses directly on thin sections. The analyses focused on cooking plates, where vertical transects were performed to assess potential thermal alterations, and on shells, to investigate mineralogical transitions between aragonite and calcite that may indicate food processing techniques and/or macroscopically invisible burning events (see Simões and Aldeias 2022).

Micro-ATR FTIR spectra were collected with a Bruker Hyperion II FTIR microscope equipped with a liquid-nitrogen-cooled LN-MCT detector and a 20x ATR objective with a germanium crystal. For each point analysis 64 scans were acquired, ranging from 4000 to 600 cm^− 1^ and with a spectral resolution of 4 cm^− 1^. The analysis spot size is 32 μm. Spectral analysis was performed with the Opus software (Bruker).

## Results and discussion

### Field evidence

#### The hearth sequence of the profile 1969–1972

The hearth sequence of the ‘second habitation area’ was exposed for approximately 7 m through a longitudinal profile (Fig. 4).

The first activities recorded consist of localised negative features dug into a natural peat layer (SU 25), which were likely related to horizontal wooden elements. At the northern edge of the profile, a finely laminated deposit, 5–6 cm thick, consisted of alternating layers of bivalve shells and charcoal lenses (SU 52). This type of accumulation was defined as shell midden (‘*chiocciolaio*’ in Italian) by Barfield et al. (1986, p. 69). Another horizontal wooden element (SU 51) was placed, probably as part of the construction of the ‘platform’ described by Barfield et al. (1986, p. 69; see Fig. 2a), followed by the deposition of a grain-supported charcoal layer (SU 53 = 57).

At the southern edge of the profile, another accumulation of calcined bivalve shells interspersed with charcoal lenses (SU 58) was recorded, 10–12 cm thick and truncated at the top by modern agricultural activities. Immediately to the north, a rubified silty-clay lens (SU 54), interpreted as a cooking plate, was identified, overlain by a finely stratified deposit of ash and charcoal (SU 55).

Later, the deliberate placement of limestone blocks appeared to separate the series of shell middens and charcoal/ash accumulations to the south from another rubified cooking plate to the north (SU 59). Near one of these blocks (SU 88), which acted as a structural boundary, the northernmost extent of another shell midden with a charcoal-rich base (SU 56) was recorded.

The sequence is sealed by a significant concentration of thermally-altered limestone blocks. However, their distribution and the thickness of the layer have been affected by previous archaeological investigations and subsequent agricultural activities, which likely lowered and reshaped the original combustion structure (compare Fig. 2a and b).

#### The new cooking plate from the 2022 excavation

The newly discovered cooking plate was a circular feature with a diameter of 1.10 m, surrounded by a ring of limestone blocks (SU 30).

The cooking plate consisted of a brownish silty-clay matrix (10YR 4/2) containing charcoal and sporadic ceramic fragments, with a notable mixture of aggregates of different colours (SU 66): reddened soil (5YR 5/8), light olive-grey silty-clay aggregates (2.5Y 5/4–4/4), and darker aggregates (7.5Y 3/1). At the base of SU 66, large carbonised wood fragments were placed in direct contact with the natural peat (SU 25; not visible in Fig. 7). Above the hearth structure, a laminated sequence of ash deposits was identified, with stratigraphic units distinguished based on the composition of the laminae (from bottom to top, SUs 64, 62, 59, 61, 6 in Fig. 7). Some layers were characterised by millimetric charcoal and sporadic shell fragments (SU 64), others by numerous light olive-grey silty-clay aggregates (SUs 62, 61), while others contained frequent shell fragments (SUs 46, 59). These laminae were also interspersed with episodes of hearth structuring, marked by the placement of limestone blocks around the perimeter (SUs 63, 65). However, these structuring events were preserved in a residual form (see Nicosia et al. 2025, pp. 57–58 for further details).

The final phase of activity at this hearth involved the deposition of an ash layer containing sporadic ceramic fragments, burnt soil aggregates, and frequent limestone fragments (SU 6), which was surrounded by a final arrangement of calcined limestone blocks (SU 30) which redefined the perimeter of plate. The deposit related to this combustion structure formed a convex morphology approximately 15 cm thick, but it has been partially truncated by modern agricultural activities.

### Micromorphology

Six SMT have been distinguished and the key micromorphological characteristics of each SMT are described in Supporting Information 1 (SI1).

#### SMT 1 – laminated lake calcareous marl

**Fig. 8:**
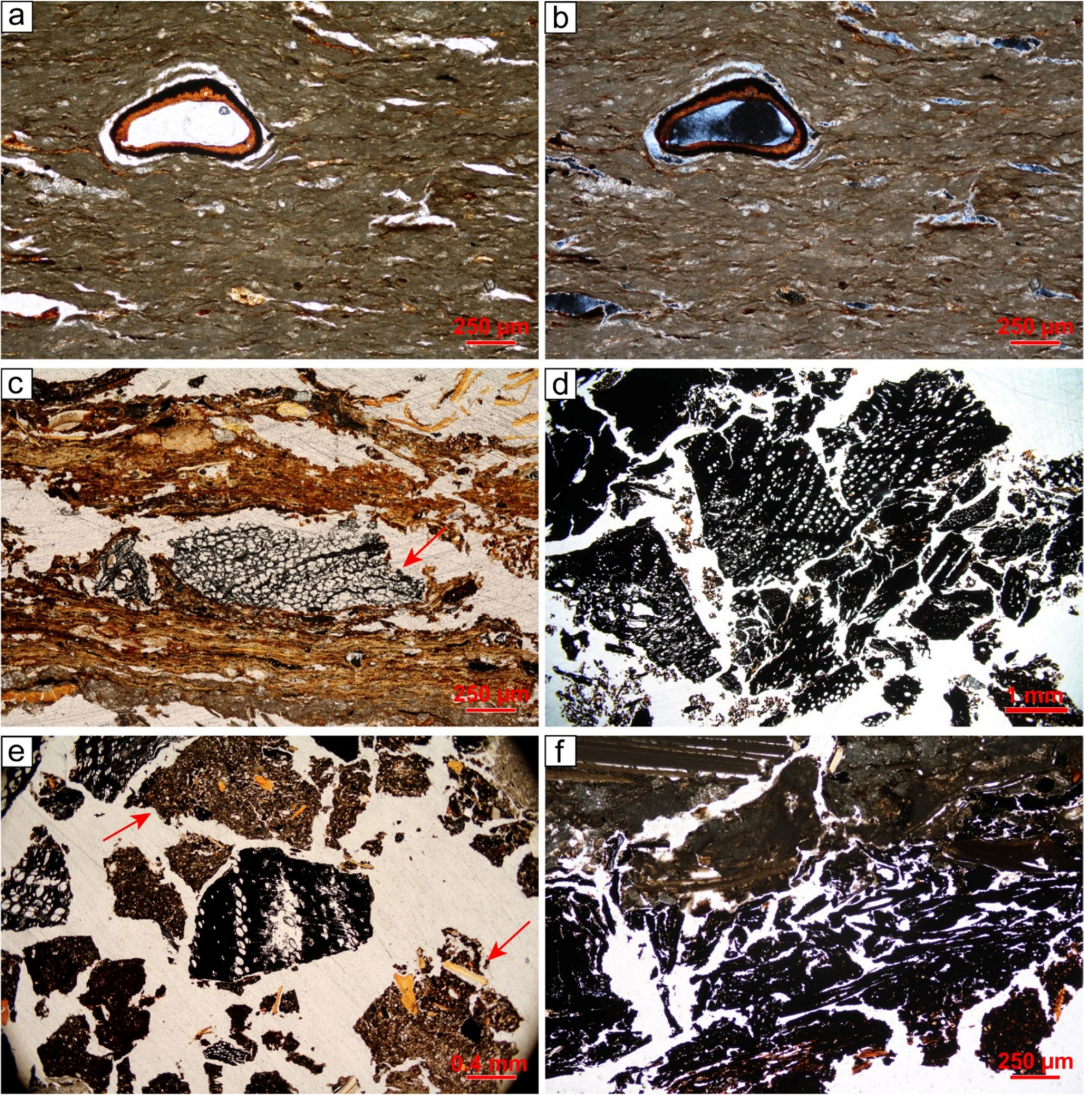
SMTs 1, 2, and 3: (**a**) SMT 1a – lake carbonate marl with numerous horizontally-lying organic tissues, PPL; (**b**) same but XPL; (**c**) SMT 2 – peat consisting of horizontally-lying brownish organic tissues intercalated with charcoal fragments (arrow). Notice the presence of scattered spheroidal organic excremental pedofeatures within voids, PPL; (**d**) SMT 3a – monogranular subunit consisting of poorly sorted charcoal fragments, PPL; (**e**) SMT 3b – isolated charcoal fragments interspersed with crumbs of organic mud (arrows) that contain bone fragments, PPL; (**f**) SMT 3c – charred peat (lower portion of the image). Notice the presence of compacted ash directly above, containing shell fragments with fissures, PPL

**SMT 1a** is characterised by a massive to weakly separated platy microstructure, with horizontal planes and very few channels. The texture is silty clay. The calcareous nature of the deposit is evident from the micritic micromass and the frequent presence of very fine sand and silt-sized sparite aggregates (Fig. 8a, b). Quartz is sporadic, while shell fragments are rare and tend to be horizontally oriented. Organic inclusions are common to frequent, comprising dark reddish brown horizontally-aligned tissues and organ residues (Fig. 8a, b). Sporadic Fe-oxide/hydroxide hypocoatings are recorded around large voids.

SMT 1a corresponds to the lake marl (SU 10) deposited during a high-stand phase of Fimon Lake (cf. Ismail-Meyer 2014; Ismail-Meyer and Rentzel 2004). During this phase the lake occupied the area where the settlement of Molino Casarotto was later established (Monegato and Nicosia in press).

**SMT 1b** shares similarities with SMT 1a but exhibits significant evidence of bioturbation, as indicated by numerous passage features (i.e., crescent coatings) and a notable increase in reworked tissue fragments and organic punctuations (10%). It marks the boundary between the peat layer on which human occupation started (SU 25; see SMT 2) and the underlying lake marl (SU 10). Bioturbation likely occurred in recent times, following the drainage of the area associated with peat quarrying activities (Vian 2022).

#### SMT 2 – peat

**SMT 2** consists of horizontally lying vegetal tissues (Fig. 8c), with very few seed pericarps, occasional fishbones, and sporadic mineral inclusions such as quartz (2%) and mica (< 2%). Notably, charcoal fragments, ranging from microcharcoal to specimens of 2.5–3 mm, are common and display a strongly expressed horizontal orientation (Fig. 8c). Sporadically, burnt soil aggregates of SMT 6d (see below) can be present. The only pedofeatures in SMT 2 are very few spheroidal organic excremental pedofeatures (Fig. 8c).

The characteristics of SMT 2 are consistent with peat accumulation in a shore context during a lake-retreat phase that preceded the human occupation. The presence of horizontally aligned charcoal fragments and burnt soil aggregates is intrusive, originating from the overlying stratigraphic units and most likely derived from trampling. Experimental studies have demonstrated that trampling can induce the downward migration of artefacts and charcoal, with movements ranging from a few to several centimetres depending on the substrate moisture and textural characteristics (Stockton 1973; Villa 1981; Villa and Courtin 1983; Gifford-Gonzalez et al. 1985; McBrearty et al. 1998; Eren et al. 2010; Driscoll et al. 2016, p. 138; Rozada et al. 2018, p. 43). The strong horizontal orientation of the charcoal fragments supports this interpretation, as trampling typically induces a preferential orientation and parallel distribution of coarser components (Miller et al. 2010; Banerjea et al. 2015; Rentzel et al. 2017; Kleijne et al. 2024). It is unlikely that the charcoal and burnt soil aggregates originated from an occupation phase which took place during peat deposition. This hypothesis can be ruled out, as radiocarbon dating indicates that peat accumulation occurred in the last centuries of the 6^th^ millennium BCE (5304–5064 cal BCE, 2σ; see Nicosia et al. 2025), and no archaeological evidence (e.g., artefacts, structures, botanical remains) from this period were identified at the site.

#### SMT 3 – charcoal-dominated units/subunits

**SMT 3a** corresponds to grain-supported charcoal layers identified in the field (SUs 53 = 57, 58) as well as other laminae composed exclusively of charcoal interspersed between cooking plates renovations (see SMTs 6e, d). This SMT is characterised by a coarse monic c/f related distribution, consisting of individual fragments of deciduous wood charcoal separated by simple packing voids (Fig. 8d). Other anthropogenic inclusions are rare to common but occur only within the single-grain charcoal layers, not in the charred laminae. These include burnt soil aggregates, burnt bones, fishbones, and horizontally lying shell fragments with evidence of snapping. Occasionally, small crumbs (up to 1.5 mm) of organic and micritic sediments (hereafter organic mud; see SMT 6b below) are present. In grain-supported charcoal layers, SMT 3a can be interpreted as the result of rake-out of domestic hearths (Mallol et al. 2017; Miller 2017; cf. microfacies – hereafter, mF – 3 in Villagran et al. 2011a). The abundance of shells and burnt fishbones highlights the importance of freshwater resources in supplementing the diet (Breglia et al. 2025). Following disposal, the deposits were trampled, as evidenced by snapped and horizontally-oriented shells (Rentzel et al. 2017; cf. mF 4 in Duarte et al. 2019). Conversely, the charred laminae embedded between hearth renovation layers can be interpreted as remnants of combustion by-products that remained in place following cleaning episodes (cf. Polisca 2025).

**SMT 3b** comprises subunits displaying a crumb microstructure, with coarse charcoal mixed with crumbs of organic mud up to 1.5 mm, burnt soil aggregates from SMT 6 d (see below), and wood ash aggregates (Fig. 8e). The anthropogenic inclusions resemble those found in SMT 3a but are notably less frequent in favour of organic mud aggregates. The b-fabric exhibits crystallitic domains (ash) alongside stipple-speckled ones (organic mud aggregates).

SMT 3b can be interpreted as trample (*sensu* Banerjea et al. 2015), representing the mixing of combustion waste from SMT 3a with sediments transported through trampling. Trample occurs due to foot traffic in wet areas, causing the redistribution of sediments that adhered to the soles of feet in either indoor and outdoor spaces (Banerjea et al. 2015, p. 98). As a result, SMT 3b is more likely to represent repeated episodes of dumping and trampling rather than a single event (cf. “occupational surfaces” in Aldeias and Bicho 2016, p. 13).

**SMT 3c** has been identified in a single instance (a subunit of SU 55, see Figs. 4 and 6) and consists of charred peat (Fig. 8f). Micromorphologically, it is composed almost entirely of blackish horizontally-oriented tissues and organs, with only sporadic silt-sized quartz.

This SMT provides evidence for the occasional use of peat as fuel at the site, a practice otherwise undocumented, given the clear preference for wood (see SMT 3a and SMT 5).

#### SMT 4 – units/subunits dominated by shell fragments

**Fig. 9:**
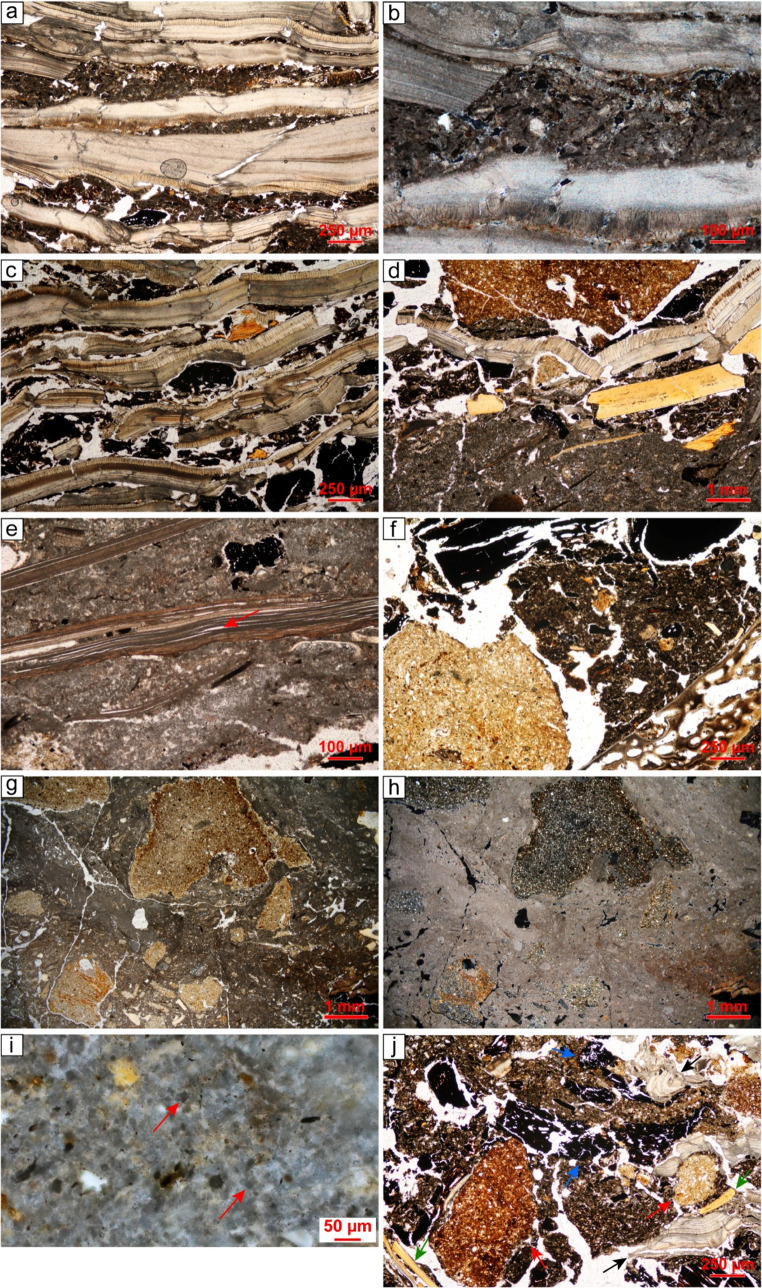
SMTs 4 and 5: (**a**) SMT 4a – horizontally lying, snapped shells with wood ash in between them. Notice the absence of fissures along the shell laminae, PPL; (**b**) detail of ‘a’, showing the ash groundmass in between the shells, PPL; (**c**) SMT 4b – horizontally lying, snapped shells with organic mud in between them. No fissures along the shell laminae are present, PPL; (**d**) SMT 5a – Subhorizontally oriented, fractured shell and bone fragments embedded within a wood ash-rich groundmass. A clay aggregate in the upper portion of the image appears to exert downward pressure on the underlying shell and bone fragments, PPL; (**e**) SMT 5a – detail of a shell fragment showing fissures along the shell laminae, PPL; (**f**) SMT 5b – ash groundmass containing charcoal (above), a burnt soil aggregate (lower left corner) and a burnt bone (lower right corner), PPL; (**g**) SMT 5c – compacted wood ash groundmass including numerous subrounded burnt soil aggregates derived from SMT 6 d, PPL; (**h**) same as ‘g’ but XPL; (**i**) SMT 5c – well-preserved wood ash rhombs (e.g., see arrows), PPL; (**j**) SMT 5 d – ash groundmass forming subangular blocky peds containing numerous charcoal (red arrows), burnt soil aggregates (blue arrows), snapped shell (black arrows), and burnt bones/fishbones (green arrows), PPL

**SMT 4a** is the most commonly identified subtype within shell midden subunits. It consists of superimposed rows of snapped and light-grey shell fragments, mixed with common horizontally-displaced coarse (up to 2 cm) charcoal from deciduous wood and very rare burnt bone fragments (Fig. 9a, b). Notably, the shells do not show fissures along laminae boundaries (cf. SMTs 5a-c). Compact wood ash is present between the shells and may contain burnt soil aggregates from SMT 6 d (see below), as well as very few burnt bone fragments, brownish organic tissues and organ residues, and fine (< 150 μm) to very fine (< 50 μm) charred/humified vegetal remains. Coarse sand-sized, rounded aggregates of brownish clay are typically present but in low quantities. The coarse inclusions exhibit an inclined orientation.

SMT 4a can be interpreted as the result of discrete tossing events following episodes of mollusc consumption (mF 4 in Villagran et al. 2011a; cf. mF 1 in Aldeias and Bicho 2016; mF 5 in Duarte et al. 2019). The absence of any evidence of colour change in PPL and XPL, as well as the lack of fissures along the laminae of shell fragments, suggests that shell processing did not involve combustion temperatures exceeding 300 °C (Villagran et al. 2011b, tbl. 3; Milano et al. 2018, p. 446; Simões and Aldeias 2022, p. 8). The ubiquitous presence of snapped shells is indicative of trampling, as observed in other shell middens located in living areas (Villagran et al. 2011a; Aldeias and Bicho 2016; Duarte et al. 2019). This fabric also suggests rapid burial, as prolonged exposure and extensive trampling would have disrupted such a fragile depositional organisation (Villagran et al. 2011a, p. 372; Aldeias and Bicho 2016, p. 11).

**SMT 4b** closely resembles SMT 4a but the inter-shell spaces are filled with organic mud (see SMT 6b) instead of wood ash. Sporadically, peat is also mixed with the organic mud, and laminae of charcoal fragments have been identified in between the shells (Fig. 9c).

The interpretation of SMT 4b aligns with that of SMT 4a. The presence of organic mud can be explained as trample or infilled material in between the shells (cf. mF 1b in Aldeias and Bicho 2016, p. 6; see also March et al. 2014, p. 29 about the infiltration of fine material in between shells).

#### SMT 5 – ash-dominated units/subunits

**SMT 5a** consists of extremely compacted wood ash, defining a massive microstructure. The groundmass also contains common microcharcoal and light-grey shell fragments, which are often horizontally displaced, snapped, and with fissure along the laminae boundaries, including cases of complete separation (Fig. 9d, e; Simões and Aldeias 2022, p. 8; Villagran et al. 2011b, tbl. 3). Rounded burnt soil aggregates from SMT 6 d (see below) are typically rare. Anthropogenic components include burnt bones and fishbones, though some fragments show no evidence of combustion. Mineral inclusions are sporadic and consist of pebble-sized limestone. Chrysophycean stomatocysts are dispersed throughout the groundmass. Notably, no pedofeatures have been documented in SMT 5a.

**SMT 5b** is compositionally similar to SMT 5a but contains a higher quantity of charcoal, charred vegetal remains, and burnt soil aggregates from SMT 6 d (see below; Fig. 9f), while shell fragments are considerably fewer. Trampling is indicated by the frequent horizontal preferential orientation of coarse components, the compaction of the deposit, and the presence of snapped inclusions (cf. Rentzel et al. 2017).

**SMT 5c** consists of extremely compacted wood ash subunits, primarily characterised by the frequent burnt soil aggregates from SMT 6 d (see below; Fig. 9g, h). Other anthropogenic inclusions, such as charcoal, burnt bones, and randomly-oriented shell fragments, are rare to very rare. Notably, shell fragments exhibit fissure along the boundaries between the laminae. The porosity is composed of vughs and channels, resulting in a vughy microstructure. As observed in SMTs 5a and 5b, wood ash is typically well-preserved (Fig. 9i), though localised recrystallisation is indicated by sparite crystals.

**SMT 5d** has been identified only in a subunit of SU 56 (see Figs. 4 and 6). It consists of wood ash forming crumbs and subangular blocky peds with few channels and vughs (Fig. 9j). The anthropogenic inclusions are similar to those reported for SMT 5a. Bones and shells are often snapped and preferentially horizontally oriented. A distinctive characteristic of SMT 5 d is the presence of silt and very fine sand quartz (< 10%).

**SMT 5e** consists of a lamina of brownish wood ash containing common charcoal fragments. It has been documented only in a subunit of SU 56, interposed between SMT 5c subunits. Similar evidence has been observed experimentally by Karkanas (2021).

The subtypes of SMT 5 can be interpreted as the result of combustion features (*sensu* Mentzer 2014). Wood was the primary fuel, as suggested by well-preserved ash pseudomorphs after calcium oxalates (Canti 2003; Gur-Arieh and Shahack-Gross 2020), while no evidence of burnt vegetal remains (e.g., chaff, blackened or molten phytoliths; the only exception is SMT 3c) or dung was observed. In general, the preservation of ash crystals and low impact of bioturbation is indicative of rapid burial in a protected (e.g., roofed, sheltered) space (Mallol et al. 2007; Gur-Arieh et al. 2014; see Friesem et al. 2017).

Micromorphological analysis confirms that these combustion features functioned as domestic cooking plates, as indicated by burnt bones and shells, likely discarded food remains. Interestingly, these shells exhibit darkening and fissures along the laminae boundaries, suggesting temperatures exceeding 300 °C (cf. section Neolithic shell middens in Italy and the relevance of a geoarchaeological approach), whereas those in SMT 4a-b (i.e., tossed shells) show no microscopic signs of heating. This difference indicates the combustion evidence likely did not occur during mollusc processing, but as a post-depositional process (i.e., subsequent fires were made on top of ash deposits that included previously discarded shells). This hypothesis is corroborated by the vughy microstructure observed in SMT 5c and the significant compaction of the deposits, as observed experimentally with relighting events by Karkanas (2021). Compaction could have also resulted from trampling, as suggested by the horizontal orientation of coarse components and snapped inclusions.

Where ash subunits are not directly above cooking plates, distinguishing in situ fire events made on rake-out layers from a sequence of rake-out deposits is challenging (see Mentzer 2014; Fig. 8 on this aspect). Experimental studies indicate that dumped deposits are characterised by an open microstructure and lack a rubified surface underneath (Miller et al. 2010; Mallol et al. 2013, 2017). Yet, subsequent trampling and compaction can obliterate these characteristics and the presence of ash as a substrate can hinder rubefaction of the underlying sediments (Aldeias et al. 2016; Karkanas 2021).

#### SMT 6 – mineral units/subunits

**Fig. 10:**
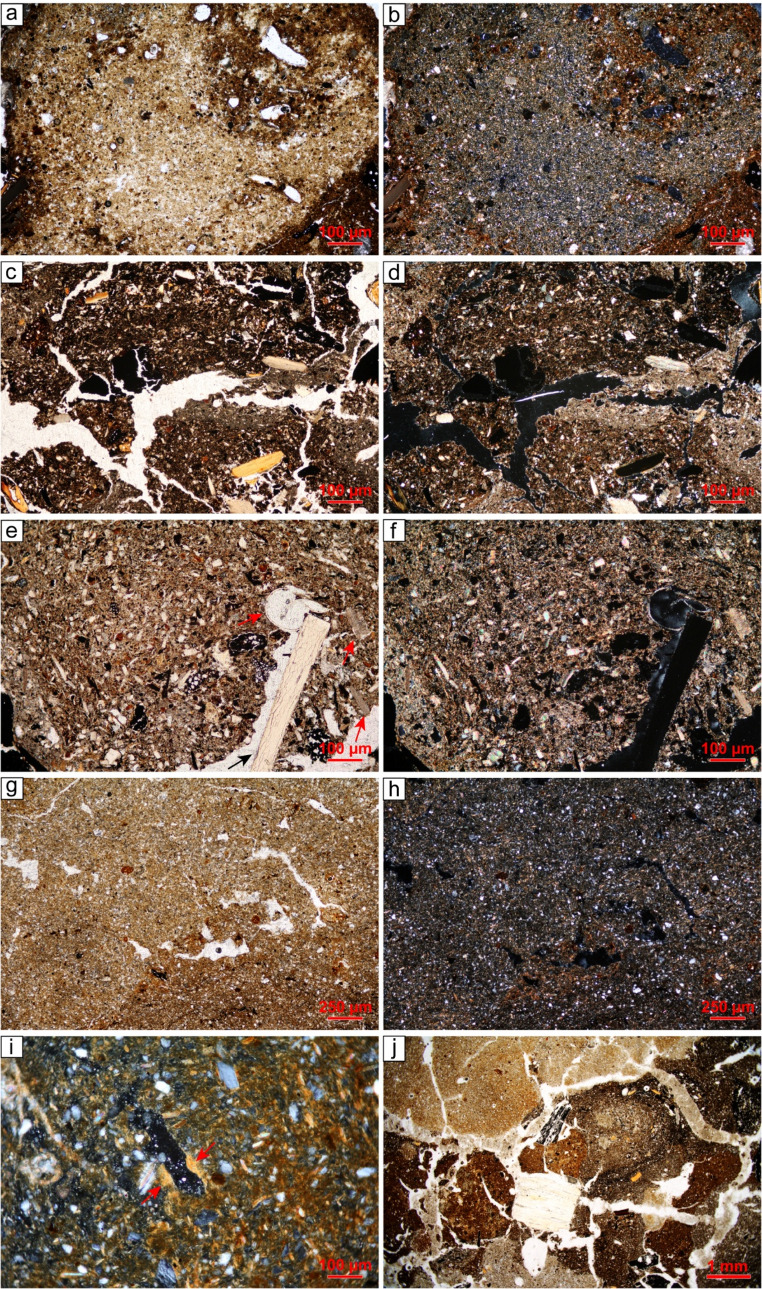
SMT 6: (**a**) SMT 6a – clay micromass with channels and silt-sized quartz, defining a porphyric c/f related distribution; (**b**) same as ‘a’ but XPL. Notice the stipple-speckled b-fabric; (**c**) SMT 6b – mud including numerous organic (blackish) punctuations and scattered charcoal, bone/fishbone and shell fragments, PPL; (**d**) same as ‘c’ but XPL. Notice the crystallitic b-fabric; (**e**) SMT 6c – calcareous silty clay including fine charred/humified organic remains, bone (black arrow) and shell fragments (e.g., red arrow), PPL; (**f**) same as ‘e’ but XPL. Notice the crystallitic b-fabric; (**g**) SMT 6d – silty clay groundmass with planar voids and vughs; **h**) same as ‘g’ but XPL. Notice the stipple-speckled b-fabric; **i**) SMT 6 d – clay micromass exhibiting a stipple-speckled b-fabric and a thin clay coating within a void (arrows), XPL; **j**) SMT 6e – subangular clay peds ranging from dark brown to yellowish brown and reddish brown, PPL

**SMT 6a** features units characterised by clay and iron oxides/hydroxide-rich micromass exhibiting a cross-striated b-fabric (Fig. 10a, b). Organic components are very few, mainly consisting of organic punctuations and very fine (< 50 μm) charred/humified tissues. Anthropogenic inclusions are rare and include charcoal fragments and randomly-oriented snapped shells reworked from the underlying layers. Bioturbation is indicated by passage features (i.e., dense, incomplete infilling).

SMT 6a characterises the modern Ap horizon (SU 2) and the layers related to the infilling of the peat quarries that were exploited up to the 1940 s in the area (SU 4). The composition aligns with the rubified soils of the Berici Hills (cf. SMT 6 d), likely the source of the material used to level area after quarrying activities. A clear indicator of this provenance is the common presence of muscovite, which is of aeolian origin (i.e., loess) and characterises these soils (Peresani 1987).

**SMT 6b** describes organic-rich, terrigenous silty clay subunits exhibiting a massive microstructure (Fig. 10c, d). The porosity consists of vertical planar voids and very few channels. The micromass is micritic and contains very rare muscovite, along with silt- and fine sand-sized calcite and quartz. Fine (< 150 μm) and very fine (< 50 μm) charred/humified organic tissues and punctuations are common, while anthropogenic inclusions are typically rare. These include snapped, light-grey shell fragments, charcoal, and silt- to medium sand-sized burnt bone fragments.

This SMT can be interpreted as trample (cf. SMT 3b) originating from the surrounding areas. This indicates damp external conditions leading to the transport of sediments due to trampling (Banerjea et al. 2015). As noted, muscovite likely derives from the rubified clayey soils of the Berici Hills. In contrast, the micritic micromass probably results from the reworking of lake marl (see SMT 1a), which is ubiquitous at shallow depths and was likely outcropping near the site.

**SMT 6c** consists of calcareous silty clay with a subangular blocky microstructure. It includes common burnt bone and fishbone fragments and rare light-grey shells (Fig. 10e, f). Mineral inclusions are common, primarily consisting of quartz, though calcite and muscovite are also present. Wood fragments are very rare.

The interpretation of SMT 6c aligns with that of SMT 6b, as these subunits likely originate from trampled material transported from the surrounding areas.

**SMT 6d** consists of silty clay sediments with a stipple-specked – locally random striated – b-fabric (Fig. 10g, h). No anthropogenic inclusions have been identified, whereas mineral components are common, primarily consisting of quartz (25–30%), and very few biotite and muscovite. Organic punctuations are very few (< 5%). A distinctive characteristic of SMT 6 d is the sporadic presence of limpid clay coatings (Fig. 10i), with some deformed clay coatings incorporated into the groundmass. Other pedofeatures consist of very few typic, disorthic, moderately impregnated Fe nodules (50–100 μm). Colour gradients have been observed, with the upper part of the units/subunits typically exhibiting a more pronounced reddish hue compared to the lower areas.

SMT 6 d corresponds to the homogeneous cooking plate structures identified along the 1969–1972 profile (see above; Fig. 4). The absence of carbonates, the identification of limpid clay coatings, along with the texture and the presence of mica, suggest that this material likely originated from the Bw/Bt horizon of the Berici Hills soils (ARPAV 2005). The reddening observed in the upper portions of the plates is likely a result of combustion processes, which induced the release of Fe oxides/hydroxides into the micromass (Canti and Linford 2000; Aldeias et al. 2016).

**SMT 6e** exhibits strong similarities with SMT 6 d but consists of well-separated subangular blocky peds, which were observed as aggregates of different colour in the field (see section The new cooking plate from the 2022 excavation). The micromass of these aggregates ranges in colour from dark brown to yellowish brown to reddish brown (Fig. 10j). However, aside from this colour variation, no other significant differences have been observed between the aggregates.

SMT 6e refers to the heterogeneous sediments used to construct the cooking plate identified in 2022 and its renovations (see above; Fig. 7). The SMT 6e subunits are either superimposed or, alternatively, separated by thin laminae of charcoal (see SMT 3a).

### Mineralogical and chemical analyses

#### Cooking plates

**Fig. 11:**
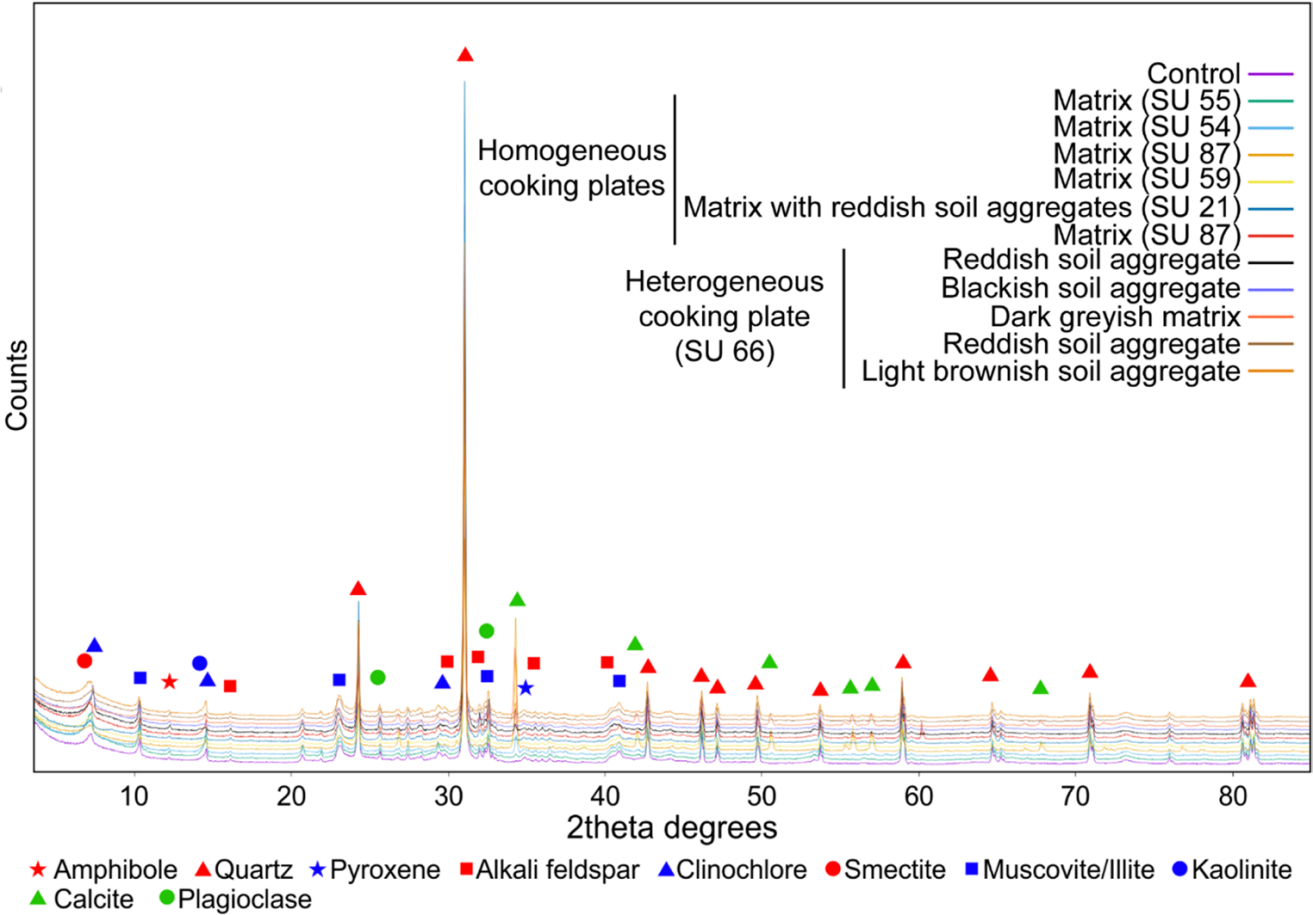
XRD spectra of the cooking plates and the control sample

In the homogeneous cooking plates (SMT 6 d), XRD patterns indicate the presence of abundant quartz, K-feldspar and clay minerals belonging to smectite and chlorite groups, kaolinite and illite/muscovite, occurring in different proportions (Fig. 11). Other mineral phases occurring in all samples are plagioclase, pyroxene, and amphibole. This mineralogical assemblage also characterises the reference sample collected from the rubified soil of the Berici Hills, confirming the use of this material to build the homogenous cooking plates (Fig. 11). Variable amounts of calcite, found in the cooking plates samples but not in the reference soil, could derive from the ash layers or infiltrated ash. Similar mineralogical composition is observed in the aggregates of different colours composing the heterogeneous cooking plate SU 66 (SMT 6e).

The mineralogical composition of cooking plates, and in particular the assemblage of clay minerals belonging to chlorite and smectite groups, compatible with that of the reference (unfired) soil sample, does not provide clear evidence of thermal alterations due firing events. Indeed, the occurrence clinochlore indicates that the heating temperatures of the cooking plates did not exceed 550 °C. Otherwise the thermal-induced phase transition of chlorite group minerals should have occurred (Maritan et al. 2006).

**Fig. 12:**
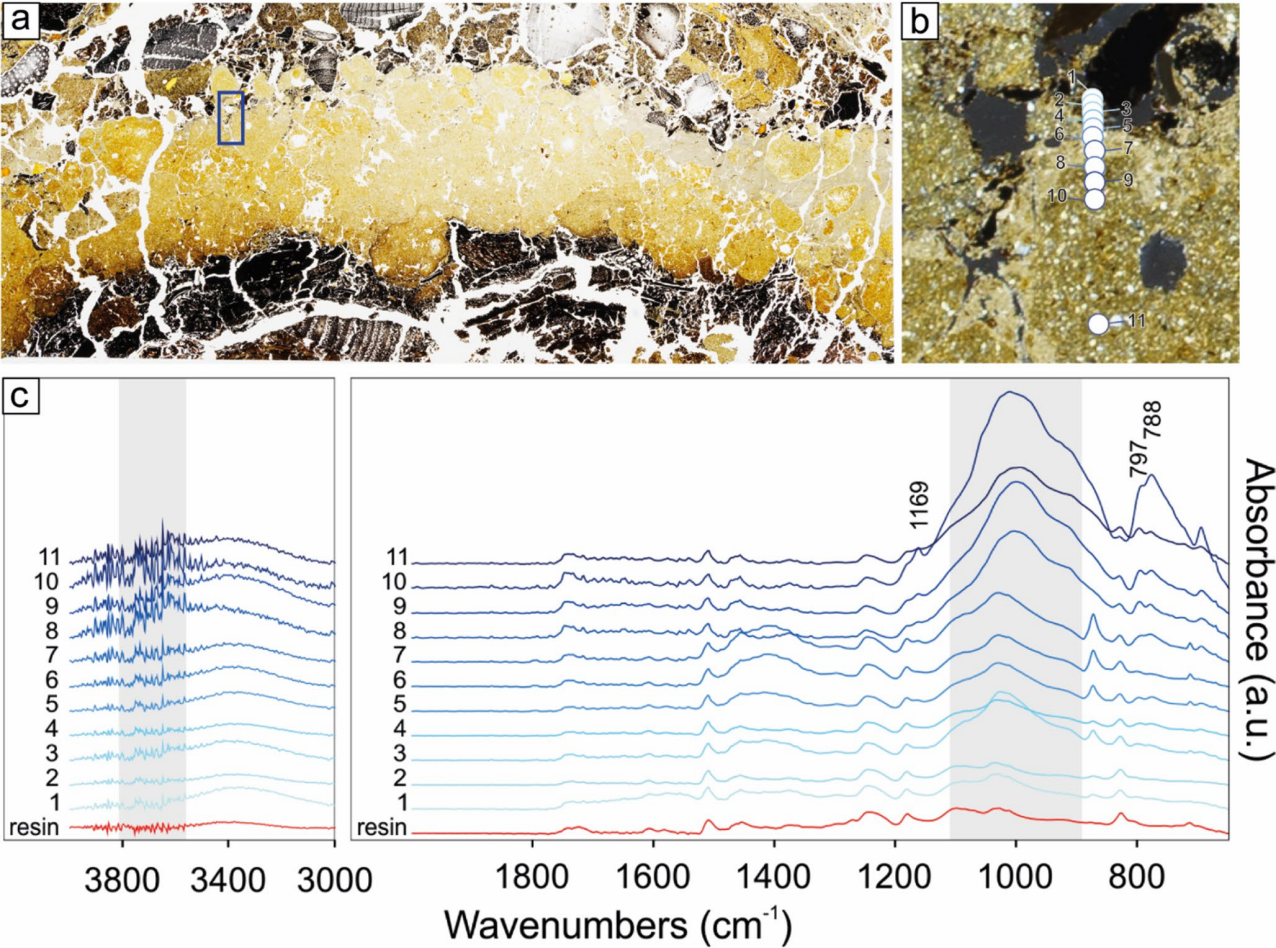
Micro-ATR FTIR spectra collected from the surface of the homogeneous cooking plate SU 54: (**a**) detail of the thin section MC22-22a (subunit 2, see Fig. 6) showing the PPL view of the cooking plate SU 54. The rectangle indicates the location of ‘b’; (**b**) position of the analysed points shown in ‘c’; (**c**) micro-ATR FTIR spectra

Given that a thermal gradient may have developed along the transect of the plate, and that phase transitions of clay minerals could have occurred in the upper portion closer to the fire, subtle mineralogical variations may have been obscured by the coarse sampling resolution used for XRD bulk analysis, which included both the base and surface of the cooking plate. To minimise potential sampling bias, XRD data were complemented with micro-ATR FTIR measurements, conducted along transects in the uppermost centimetres of the plates at a higher spatial resolution than that employed for XRD (Fig. 12). Micro-ATR FTIR results are consistent with those obtained from XRD analyses, indicating neither thermal alteration in the upper surface of the cooking plate nor significant changes in spectral features along the analysed transect. FTIR spectra show the characteristic bands of quartz (797, 788, 1169, 695 cm^− 1^), calcite (711, 871 and a broad contribution at ca. 1400 cm^− 1^), a complex region of overlapping bands in the range 900–1100 cm^− 1^ (with the superimposition of Si-O stretching bands at 1000–1100 cm^− 1^ of silicates and Al2OH bending modes at 915 cm^− 1^ occurring in dioctahedral smectites and other clay minerals; Madejová 2003) overlapping to the complex spectral features of the epoxy resin, embedding the thin sections. Moreover, despite the low sensitivity of micro-ATR FTIR and the high signal noise at high wavenumbers, all spectra show a hump at approximately 3620 cm⁻¹. This feature is attributable to the O–H stretching mode of structural hydroxyls in smectites and provides clear evidence that these layers were not exposed to high temperatures (> 700 °C), which would have caused the irreversible dehydroxylation of smectites (Malhotra and Ogloza 1989). Humps in the range 3670–3800 cm^− 1^ are compatible with the occurrence of kaolinite, clinochlore and other clay minerals.

#### Mollusc shells

**Fig. 13:**
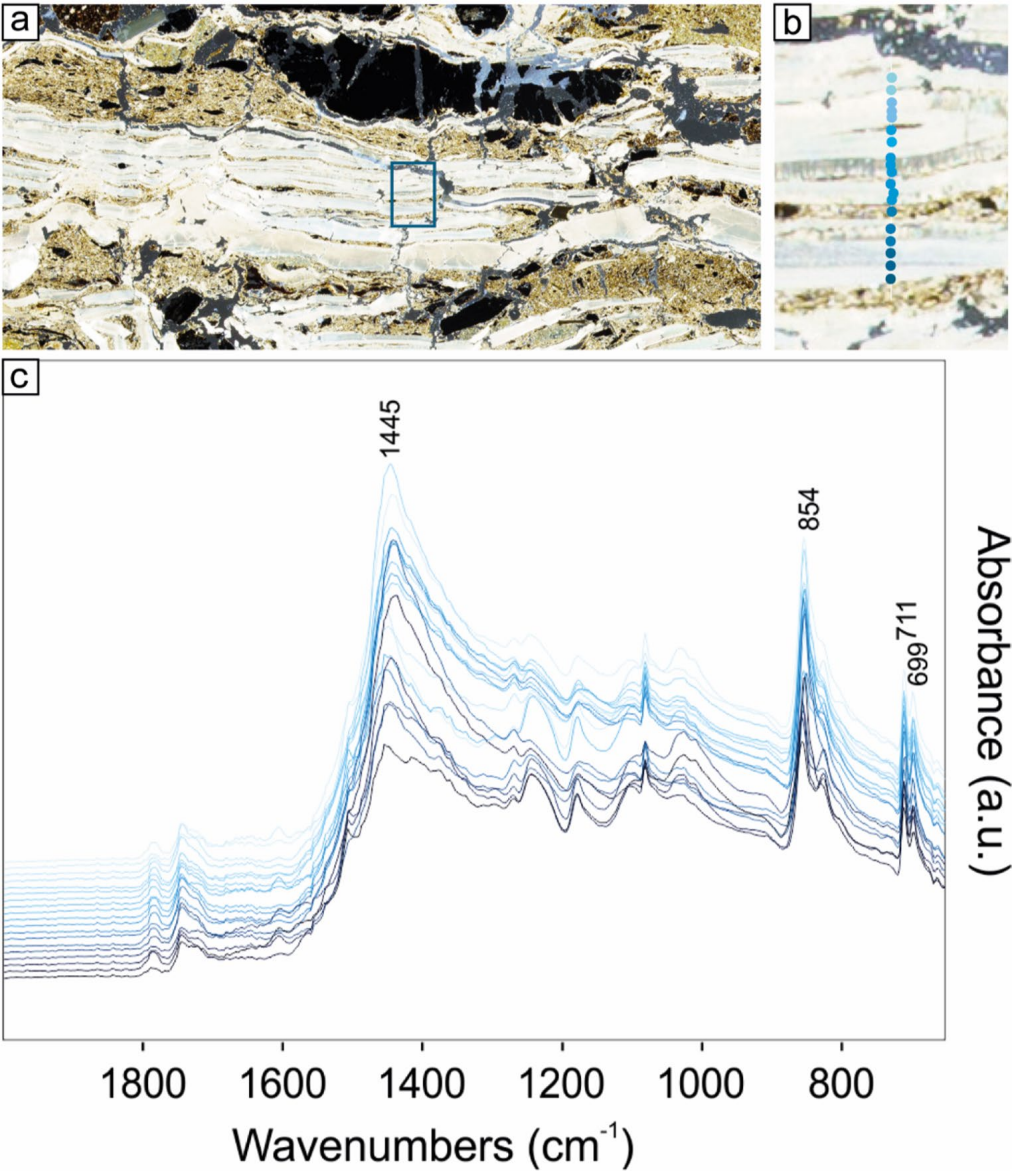
Micro-ATR FTIR spectra collected from tossed shells (SU 54): (**a**) detail of the thin section MC22-31 (subunit 5, see Fig. 6) showing the XPL view of the subunit of tossed shells (SMT 4a). The rectangle indicates the location of ‘b’; (**b**) position of the analysed points shown in ‘c’; (**c**) micro-ATR FTIR spectra

Unionid shells, like those found in the shell middens of Molino Casarotto, are mineralogically composed of aragonite and consist of an outer prismatic layer and an inner nacreous layer (Checa and Rodríguez-Navarro 2001). Micro-FTIR analyses of the tossed shells (SMT 4 subunits) only reveal vibration peaks at 854 cm^− 1^ (ν_2_), 1445 (ν_3_), and a doublet at 712 and 699 cm^− 1^ (ν_4_), which are consistent with the exclusive presence of aragonite in all the shells (Fig. 13; cf. Simões and Aldeias 2022).

**Fig. 14:**
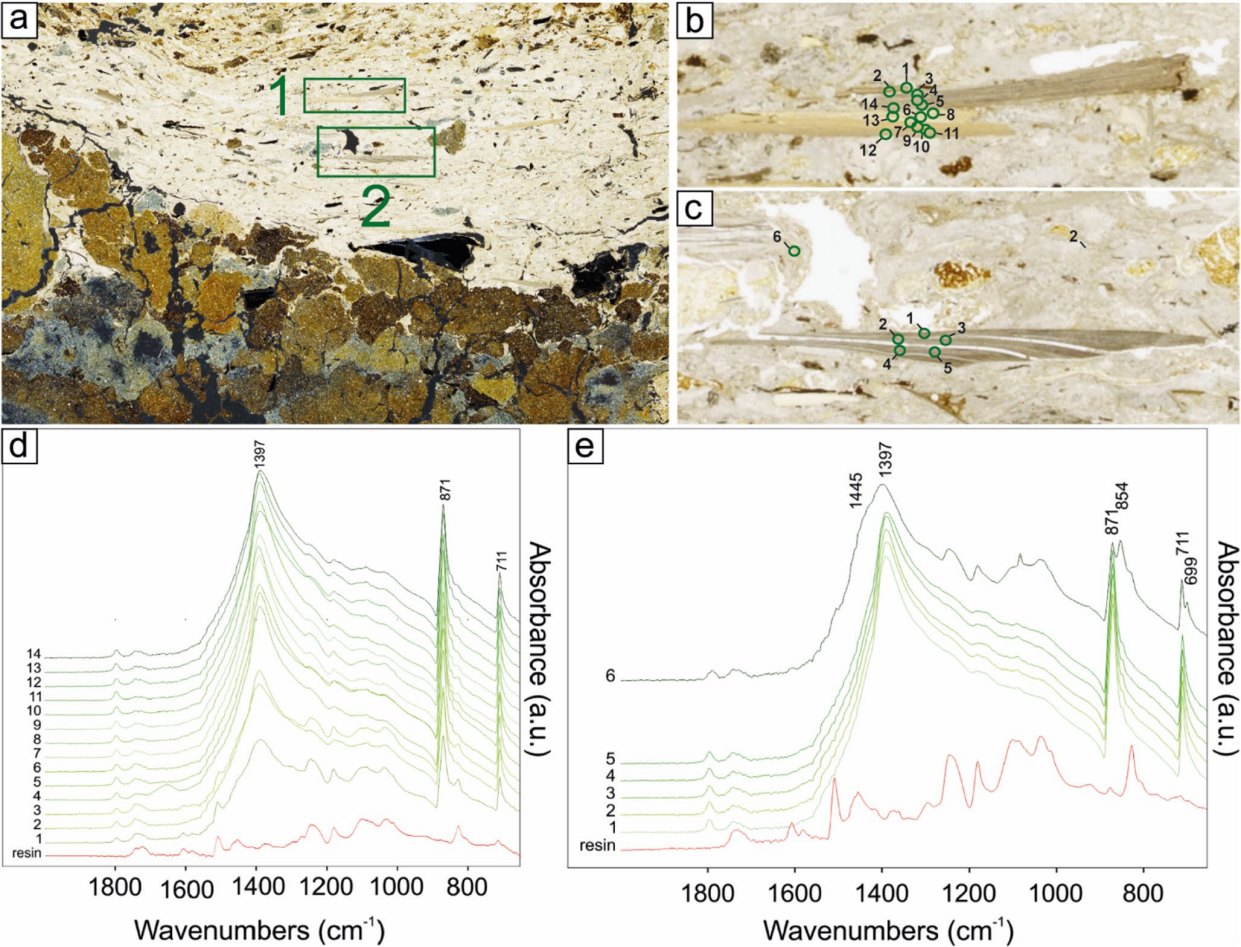
Micro-ATR FTIR spectra collected from shells within an ash subunit derived from in situ combustion (SU 54): **a**) detail of the thin section MC22-39a (subunit 2, see Fig. 7) showing the PPL view of the ash subunit including numerous shells with fissures along the boundaries between the laminae (SMT 5a). The rectangles indicate the location of ‘b’ (n. 1) and ‘c’ (n. 2); **b-c**) position of the analysed points shown in ‘d’ and ‘e’, respectively; **d-e**) micro-ATR FTIR spectra

Conversely, the shells embedded in situ combustion or hearth rake-out layers (SMT 5 subunits) typically display a complete transition of the shells to calcite, as indicated by the presence of its characteristic peaks (ν3, ν4 and ν2 peaks at 1397 cm − 1, 871 cm^− 1^, 711 cm^− 1^, respectively; see Weiner 2010) and the absence of the aragonite peaks (Fig. 14). This suggests combustion temperatures around 400–500 °C (Maritan et al. 2007, p. 531; Milano et al. 2018, p. 448; Simões and Aldeias 2022, p. 8). In some cases, instead, shells show mixtures of aragonite and calcite polymorphs, testifying combustion temperatures above 250 °C but not sufficient for a complete transition (Fig. 14; Simões and Aldeias 2022).

### Archaeological and behavioural implications

#### Continuous and sheltered sedimentation

Micromorphological analysis of both raked-out and in situ combustion features indicates a repeated and continuous domestic use of the space, with no evidence of occupational breaks that would be signalled by erosive processes, natural sedimentation, or localised weathering and bioturbation (cf. Balbo et al. 2010). This has significant archaeological implications as previous research could not rule out whether the site was occupied all-year-round or seasonally as a hunting/foraging camp site (Bagolini 1984, p. 387).

In all combustion features, the presence of laminated deposits of wood ash crystals with limited evidence of recrystallisation suggests that deposition occurred relatively quickly within a sheltered or roofed environment. In open-air settings, ash is typically subject to rapid dispersal by wind or dissolution by rain, as demonstrated by geo-ethnoarchaeological research (Shahack-Gross et al. 2004, p. 1401; Mallol et al. 2007; Miller et al. 2010, p. 35; Friesem et al. 2017). A rapid burial and limited post-depositional reworking are also suggested by the shell midden subunits, where most shells remain articulated (cf. mF 4 in Villagran et al. 2011a, p. 372). However, the presence of snapped inclusions indicates the area was trampled (Kleijne et al. 2024).

#### Insights into subsistence practices

No traces of cereal processing (e.g., phytoliths, charred/ashed chaff) were identified through micromorphology. This finding is consistent with the results of the archaeobotanical analysis from the 2022 excavation, which recovered only nine cereal grains despite the large volume of sediment processed (166 L; Breglia et al. 2025).

In addition to shell gathering, zooarchaeological analysis also documented fishing activities at the site. However, despite wet-sieving 162 L and 154 L of sediment through 1 mm and 2 mm meshes respectively, only 70 fishbones were recovered from the 2022 excavation (Breglia et al. 2025). In contrast, fish remains are frequently observed in thin section, particularly within charcoal-rich (SMT 3) and ash-rich (SMT 5) subunits, where they appear as elongated spines measuring up to 300–400 μm in length. These results suggest that a comprehensive analysis of fishbone assemblages would require sieving with a much finer mesh (i.e., < 300 μm), a method that is extremely time-consuming and often impractical when large sediment volumes are involved. Micromorphological analysis therefore represents a valuable tool for detecting fish remains in the sedimentary record, even when conventional zooarchaeological approaches underestimate their presence. At Molino Casarotto, evidence of such a sampling bias suggests a more prominent role of fishing in the settlement’s subsistence economy than previously supposed.

This interpretation is fully consistent with a site economy largely based on hunting and gathering activities (Breglia et al. 2025; Bagolini et al. 1973, p. 210). Since a seasonal camp function can be ruled out, the absence of evidence for cereal cultivation and the limited role of herding should be understood as the outcome of a deliberate economic choice. It can be thus proposed that the perilacustrine environment and the nearby Berici Hills provided the inhabitants with sufficient resources (e.g., wild game, fish, shells, wild fruits, with water chestnut likely serving as the staple food) to avoid cultivating and herding (cf. Karg 2006; Tolar et al. 2011; Borojević 2012).

#### Hearth maintenance, mollusc cooking practices, and shell disposal

Considering combustibles, wood was the dominant fuel used at the site, with only occasional evidence of charred peat. Cooking plates were typically renovated several times, as indicated by slightly rubified surfaces or microscopic laminae of charcoal that escaped cleaning. Therefore, the combustion by-products identified on top of the heterogeneous cooking plate SU 66 likely represent its final phase of use rather than its entire accumulation. At Molino Casarotto, the presence of in situ and raked-out ash deposits indicates that the cooking plates functioned not only for cooking through heat conduction but also as simple hearths (cf. the section Neolithic combustion features in Italy). This is particularly evident in the case of the cooking plate SU 66 (Fig. 7). This interpretation is further supported by a fence-like alignment of vertical posts to the north of this structure, possibly functioning as a windbreak (Fig. 3). The importance of wind control in relation to hearth use has been emphasised by ethnographic research (e.g., Binford 1978, p. 349).

Furthermore, the thick, in situ, laminated, and compacted ash deposit observed above SU 66 (Fig. 7) indicates that cooking plates were not cleaned after every use and that relighting occurred (Mallol et al. 2013, p. 20; Karkanas 2021). Given the insulating properties of ash (Aldeias et al. 2016; Canti 2025), this can explain the absence of mineralogical evidence of significant thermal alteration of the cooking plates, as shown by p-XRD and micro-AFT FTIR data.

Relighting events may also explain why thermally altered shells were found exclusively within ash-rich layers (SMT 5), whereas those in shell middens (SMT 4) show no signs of alteration. The occurrence of this polymorphic transition only in ash-rich subunits suggests that the alteration is unrelated to mollusc processing prior to consumption. This interpretation is further supported by the fact that the fissures observed in altered shells require temperatures exceeding 300 °C to form, while roasting above 200 °C would render the molluscs inedible (Simões and Aldeias 2022, p. 9). In behavioural terms, the distribution of some heated shells within rake-out deposits and their deposition in separate middens is consistent with Meehan’s ethnographic descriptions of shellfish consumption practices among the Anbarra Australian Aborigines (“A few shells usually get tossed around the hearth but they are normally left for the time being in discrete piles where they have been consumed, many pairs of valves remaining attached by gristle” in Meehan 1982, p. 87). From a methodological standpoint, this information has significant implications for oxygen isotope analysis, suggesting that only shells from midden contexts are likely to preserve unaltered isotopic ratios (Milano et al. 2016, 2018; Müller et al. 2017).

Considering the mollusc processing technique, the close spatial association between shells and cooking plates suggests that fire or heat was used, rather than cracking, perforating, or shucking techniques (Waselkov 1987, p. 100). The cooking of shells could have occurred either through boiling or roasting at low temperature (below 200 °C), i.e. techniques that do not leave significant traces in archaeological deposits (Aldeias et al. 2019). The presence of burnt limestone blocks associated with combustion features and shell middens may indicate their role in the processing. Ethnographically, heated stones – also referred to as ‘hot stones/rocks’ or ‘cook-stones’ (Brink and Dawe 2003; Thoms 2008) – were used to steam molluscs laying them directly upon the stones (Waselkov 1987, tabl. 3.1). Such technique could also have been used to steam other types of products, such as meat, fish, or vegetables (Lovisato 1884, p. 142; e.g., Heizer 1975, p. 30; Thoms 2003). Alternatively, stones may have been heated in a fire, then immersed into water to induce boiling and cook foodstuff (Thoms 2003). The available data do not allow us to distinguish between low-temperature roasting – typically a quick process used to open the shells (i.e., few minutes; cf. Meehan 1982, p. 87; Waselkov 1987, pp. 103–105) – or boiling.

## Conclusion

Compared to the large number of known open-air Neolithic sites in northern Italy (cf. Pessina and Tiné 2022), relatively few intra-site deposits have been investigated through micromorphological analysis (Bernabò Brea et al. 2008; Pedrotti et al. 2015; Pescio et al. 2017; Degasperi et al. 2019; Tasca et al. 2019). Previous studies have primarily focused on pit features, fill accumulation processes, or individual combustion structures. This work thus represents the first extensive micromorphological investigation that integrates both domestic deposits and their associated cooking plates.

The well-preserved, finely laminated sequences at Molino Casarotto enabled high-resolution sediment analyses that revealed significant insights on the use of space and daily activities at the site:

1. Despite the limited architectural evidence, the combustion structures appear to have been used within a sheltered space for domestic purposes such as cooking meat, molluscs, and fish. In this respect, micromorphological analysis suggests that fish may have played a greater role in the diet than what previous zooarchaeological analysis alone would indicate.
2. The continuous sedimentation highlights that the site was likely occupied throughout the year, thereby excluding a seasonal use associated solely with hunting and gathering practices. This aspect is crucial for correctly interpreting the site’s subsistence economy and its significance within the Middle Neolithic archaeological framework (see Breglia et al. 2025 for further details).
3. Micro-FTIR analyses on the shells suggest that the molluscs were likely boiled or roasted at low temperatures, possibly using cook-stones, as indicated by the absence of fissures and of aragonite-to-calcite transformation in shells from the middens. This practice would have allowed the shell to be opened without the risk of charring the mollusc inside. In contrast, significant thermal alteration has been observed in the shells recovered from combustion features due to post-depositional heating. As a result, future isotopic analyses aimed at investigating paleoenvironmental conditions and shell-gathering seasonality should rely solely on midden-derived shells, which represent the most reliable archive. Differential preservation of shells across deposit types should be taken into account at other sites where similar analytical approaches are planned.
4. Cooking plates were periodically cleaned, though not on a daily basis, as indicated by evidence of relighting and by the presence of thick ash accumulations above them. These deposits likely reduced heat transfer, preventing the plates from reaching combustion temperatures above 700 °C, as demonstrated by XRD and micro-FTIR analyses. Another – and not mutually exclusive – explanation for the absence of significant thermal alteration in the cooking plates may relate to the use of cook-stones to heat the plates or to boil/roast the shells. This practice would have avoided lighting fires directly on top of the plates.

These observations, made possible by a micro-contextual approach, would have been difficult to discern through field observations alone. This study therefore demonstrates the significant potential of geoarchaeological methods in reconstructing everyday life and food preparation practices in Neolithic contexts.

## Supplementary Information

Supplementary File 1 (XLSX 16.7 KB)

## Data Availability

Data is provided within the manuscript or supplementary information files.
